# Disentangling drivers behind fungal diversity gradients along altitude and latitude

**DOI:** 10.1111/nph.70012

**Published:** 2025-02-25

**Authors:** Florian Barbi, Tijana Martinović, Iñaki Odriozola, Antonin Machac, Andrea Moravcová, Camelia Algora, Dalibor Ballian, Sebastian Barthold, Vendula Brabcová, Sandra Awokunle Hollá, Zander Human, Hojka Kraigher, Jelena Lazarević, Clementine Lepinay, Lenka Mészárošová, Daniel Kumazawa Morais, Nikolai Nikolov, Ella Thoen, Vojtěch Tláskal, Tomáš Větrovský, Petr Baldrian, Petr Kohout

**Affiliations:** ^1^ Laboratory of Environmental Microbiology Institute of Microbiology of the Czech Academy of Sciences Videnska 1083 Prague 142 20 Czechia; ^2^ Department for Forest Physiology and Genetics Slovenian Forestry Institute Večna pot 2 1000 Ljubljana Slovenia; ^3^ Faculty of Science Charles University Albertov 6 Prague 128 40 Czechia; ^4^ Faculty of Forestry University of Sarajevo Zagrebačka 20 71000 Sarajevo Bosnia and Herzegovina; ^5^ Academy of Sciences and Arts of Bosnia Bistrik 7 71000 Sarajevo Bosnia and Herzegovina; ^6^ Nationalpark Harz – Außenstelle Oderhaus Oderhaus 1 37444 Sankt Andreasberg Germany; ^7^ Biotechnical Faculty University of Montenegro Mihaila Lalića 15 81000 Podgorica Montenegro; ^8^ Plant Pathology Unit INRAE 67 allée des chênes 84143 Montfavet France; ^9^ Norwegian College of Fishery Science, UiT The Arctic University of Norway PO Box 6050, Langnes N‐9037 Tromsø Norway; ^10^ Vitosha Nature Park 17 ‘Antim I’ Str 1303 Sofia Bulgaria; ^11^ Department of Biosciences University of Oslo PO Box 1066, Blindern 0316 Oslo Norway

**Keywords:** altitudinal and latitudinal gradients, biogeography, climate, ectomycorrhizal fungi, fungal diversity, join species distribution models, root endophytic fungi, saprotrophic fungi

## Abstract

- Gradients in species diversity across elevations and latitudes have fascinated biologists for decades. While these gradients have been well documented for macroorganisms, there is limited consensus about their universality, shape and drivers for microorganisms, such as fungi, despite the importance of fungal diversity for ecosystem functions and services.
- We conducted a comprehensive survey of fungal species richness in forests across 17 elevational transects along a latitudinal gradient covering the continental scale of Europe.
- Diversity patterns along elevational and latitudinal gradients differed among fungal ecological guilds. Diversity of saprotrophs declined with elevation while ectomycorrhizal (ECM) fungal diversity peaked in mid‐elevations. Moreover, the diversity of root endophytic fungi increased with latitude but did not change with elevation. Bayesian species distribution modeling suggests that fungal diversity is structured by deterministic rather than stochastic drivers. Importantly, ECM fungal diversity pattern persists even after accounting for the effects of environmental conditions.
- These results suggest that environmental conditions differentially shape the diversity of fungal guilds along elevational and latitudinal gradients, but this goes beyond soil and climatic factors in the case of ECM fungi. This study paves the way toward a better understanding of fungal diversity gradients across elevations and latitudes, with possible implications for macroecological theory, conservation and management.

## Abstract 2

See also the Commentary on this article by Kennedy & Smith, **247**: 11–13.

## Introduction

Ever since the work of Johann Reinhold Forster and Alexander von Humboldt, biologists have been fascinated by the striking gradients in species diversity across latitudes (Forster, 1778) and elevations (von Humboldt & Bonpland, 2013). While these diversity gradients and their underlying mechanisms have received significant attention and seem ubiquitous in vertebrates, insects and plants (Lomolino, 2001; Willig *et al*., 2003; Hillebrand, 2004; Rahbek, 2005; Mannion *et al*., 2014; Liang *et al*., 2022; Sabatini *et al*., 2022), investigations have been comparatively less extensive for microscopic organisms, such as fungi, regardless of their ubiquity and importance in ecosystems.

Fungi are one of the most diverse groups of organisms, with an estimated 2–6 million species (Baldrian *et al*., 2022, 2023; Niskanen *et al*., 2023) and ecologically classified based on the resources they use. As major decomposers of organic matter, as well as mutualists or parasites of plants, fungi significantly influence plant primary production and carbon sequestration (Netherway *et al*., 2021). While climate and interaction with other decomposers (ants and termites) likely stand behind the diversity patterns of free‐living saprotrophic (SAP) fungi (Crowther *et al*., 2012), diversity of biotrophic mycorrhizal and pathogenic fungi correlates with the abundance (Tedersoo *et al*., 2014; Soudzilovskaia *et al*., 2019; Steidinger *et al*., 2019) and diversity (Van der Heijden *et al*., 1998, 2008; Bagchi *et al*., 2010) of their host plants. Therefore, the effect of different environmental variables on species diversity and distribution varies to a large extent according to the fungal ecological group (Martinović *et al*., 2021; Odriozola *et al*., 2024).

Diversity of fungi along various environmental gradients has been previously addressed globally for latitude (Tedersoo *et al*., 2012; Treseder *et al*., 2014; Větrovský *et al*., 2019) and locally for elevation (Coince *et al*., 2014; Miyamoto *et al*., 2014; Geml *et al*., 2022). Despite a recent fast‐increasing number of studies, the conclusions regarding the patterns and their drivers have been characterized as ‘consistently inconsistent’ (Hendershot *et al*., 2017). Consequently, it remains unclear whether fungi mirror diversity gradients known from macroorganisms or exhibit distinct patterns, such as having the highest diversity toward the gradient edges, mid‐elevation peaks or no general trends at all. Recent large‐scale studies clearly showed that fungal diversity does not always decrease with latitude (Větrovský *et al*., 2019; Mikryukov *et al*., 2023). Similarly, the trend of fungal diversity along the elevation gradients can range from monotonic negative (Bahram *et al*., 2012; Shigyo & Hirao, 2021) to positive (Ni *et al*., 2018; Shigyo & Hirao, 2021) or even unimodal (Ogwu *et al*., 2019; Geml *et al*., 2022). These mixed results could stem from the differences in the methodological approaches of previous studies. However, they may also be caused by different roles of environmental drivers (e.g. regional climate, soil chemistry, the geometric constraints – that is physical boundaries that limit the range of species – of the individual mountain ranges or biological factors such as differences in the natural history of fungal guilds) in structuring fungal diversity along latitudinal and elevational gradients. While geometric constraints should affect fungal diversity along small‐scale elevational gradients, environmental conditions should likely play a more important role along latitude.

In this study, we explored elevational and latitudinal gradients in fungal diversity and species‐level responses to these gradients. For this purpose, we present a unique survey covering forests, dominated by ectomycorrhizal (ECM) tree species, along 17 elevation transects across Europe. We used mixed models to assess gradients in diversity for the entire fungal community (further referred to as ‘whole fungi’ (FUNGI)) as well as specific fungal guilds (ECM, SAP and root endophytic (REND) fungi (i.e. fungi that are considered as nonobligatory symbiotic fungi with plant roots, root endophytes and ericoid mycorrhizal fungi)). Additionally, we used joint species distribution modeling to capture the responses of individual species both to latitude and to elevation. Through this approach, we aimed to disentangle the architecture of diversity patterns by verifying if individual species occurrences reflected general diversity. To identify underlying mechanisms behind these diversity patterns, we modeled species richness of the different fungal guild along elevation and latitude after accounting for the effect of environmental variables. Finally, we employed a random forest modeling approach to identify environmental variables that drive fungal species richness along the studied gradients. We hypothesize that, if fungal species richness patterns differ between elevational and latitudinal gradients, distinct drivers should shape fungal diversity along these gradients. Furthermore, given the differences in lifestyle and climatic niches among fungal guilds, we hypothesized that diversity patterns would vary between fungal guilds.

## Materials and Methods

### Study sites

**Fig. 1:**
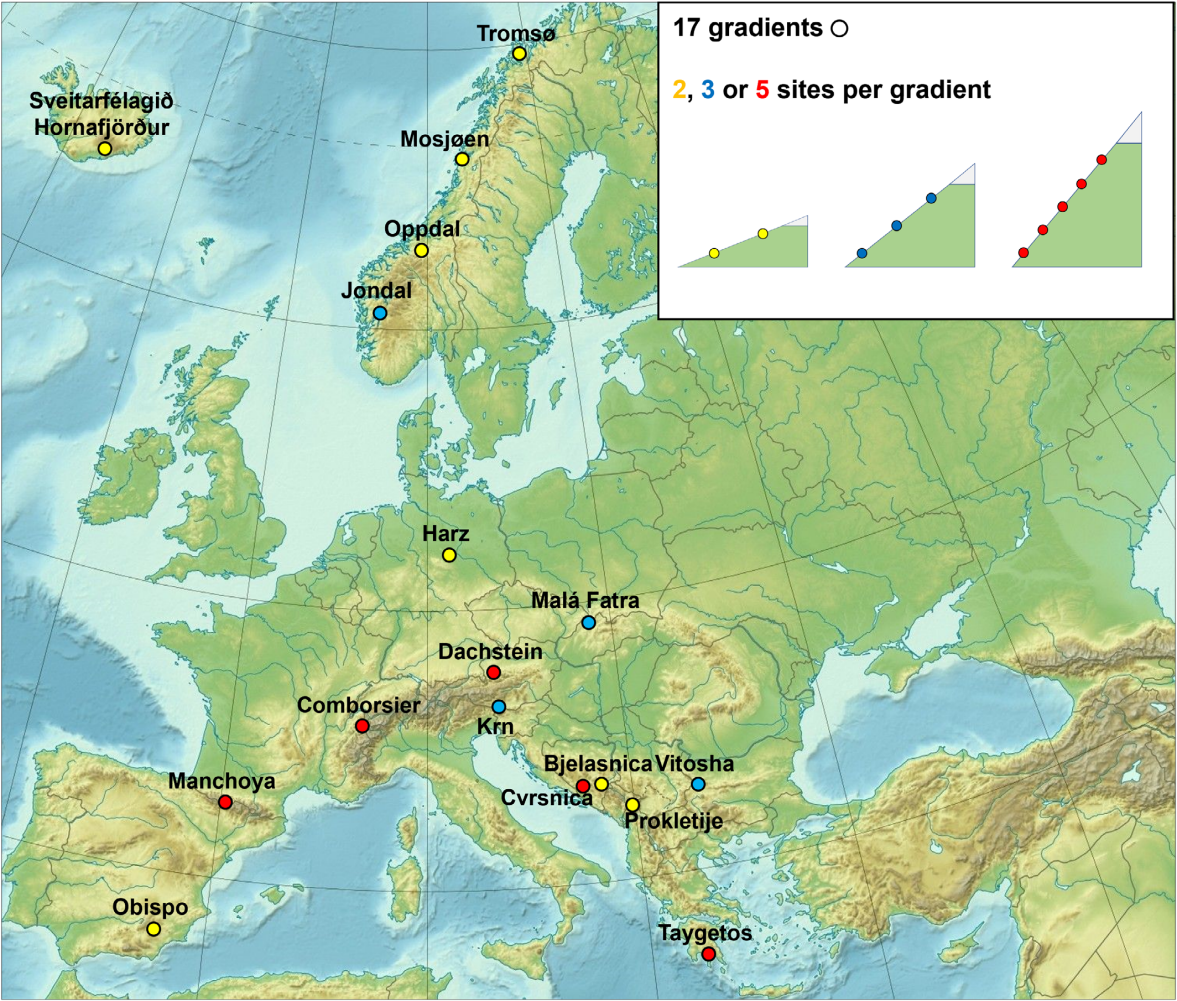
Map of the 17 locations where sampling was performed along mountain slopes. The colors indicate the number of sampled sites in each location (yellow, 2 sites; blue, 3 sites; red, 5 sites).

Soil was sampled in pure ECM‐dominated forests with a very homogenous diversity of tree species (no more than two species per site) at 17 altitudinal transects across Europe (Fig. 1; Supporting Information Table S1) between 2018 and 2019. Sampling took place in two, three or five elevational levels with three replicated plots per elevation, in a total of 159 plots. The sampling started between 100 and 200 elevational meters below the tree line, to avoid the effect of the ecosystem's ecotone, and the same elevational distance separated each level. The plots within the same elevation level were selected to represent the dominant local forest vegetation. At each plot, five 4‐cm diameter soil cores were collected using a different corer for each core: one central core and four additional cores located 2 m north, south, east and west of the central core (Martinović *et al*., 2021). The corers were thoroughly washed before being reused for sampling on different days, to prevent cross‐contamination. Soil cores were stored at 4°C and processed within 24 h after collection. Plant litter was removed as well as the soil below 10 cm depth. Stones and large roots were removed and the content of the five cores from each plot was mixed to obtain a composite sample. The soil was sieved through a 5‐mm mesh sieve, which was washed with water and ethanol between each composite sample. Samples were then freeze‐dried on an in‐house lyophilizer (Labio a.s., Prague, Czechia) and stored at −20°C until further analyses.

### Soil chemistry analysis

Soil pH, from 3.71 to 7.79, was measured in deionized H_2_O (1 : 10 w/v sample : liquid ratio) using a WTW Multilab 540 pH instrument (Gemini BV, Apeldoorn, the Netherlands). Organic carbon (C) concentration was analyzed using sulphochromic oxidation, whereas total nitrogen (N) concentration was analyzed on a CHN Carlo Erba EA 1108 analyzer (Carlo Erba, Milano, Italy). Total P (P) was extracted from the soil samples using the Olsen method (1 : 100 w/v sample : extraction agent ratio) and measured spectrophotometrically at 630 nm (UV‐400 Unicam; Thermo Scientific, Waltham, MA, USA). All soil chemistry analyses were performed in an external laboratory of the Institute of Botany of the Czech Academy of Sciences (Průhonice, Czech Republic) (Table S1).

### Molecular analysis

Total genomic DNA was extracted in triplicate using 250 mg of freeze‐dried soil samples, following a modified Miller method (Sagova‐Mareckova *et al*., 2008). DNA extracts were cleaned using a Geneclean Turbo Kit (MP Biomedicals, Solon, OH, USA) and pooled before subsequent PCRs. The fungal ITS2 region was amplified in triplicate from soil DNA, using gITS7 (Ihrmark *et al*., 2012) and ITS4 (White *et al*., 1990) primer pairs. Both the forward and reverse primers were tagged with sample‐specific molecular identifiers (MID). Each soil DNA sample was amplified in triplicate to reduce polymerase chain reaction (PCR) biases. More details about PCR protocols and conditions can be obtained from Moravcová *et al*. (2023). Amplicon triplicates were subsequently pooled, purified using a MinElute kit (Qiagen) and quantified with a Qubit™ dsDNA BR Assay kit (Thermo Fisher Scientific). Sequencing libraries were prepared using the TruSeq PCR‐free Kit (Illumina, San Diego, CA, USA) and sequenced in‐house using Illumina MiSeq (2 × 250).

### Bioinformatic data processing

Fungal sequence data were processed using the Seed 2.0 pipeline (Větrovský *et al*., 2018). Briefly, Fastq‐join (Aronesty, 2013) was used for joining the pair‐end reads. Quality filtering was performed with the mean quality score of 30 as a cutoff and sequences were demultiplexed according to the matching MIDs on forward reverse reads. The ITS2 region was extracted using ITSx v.1.0.8 (Nilsson *et al*., 2010) before further processing. Following the removal of chimeric sequences, the remaining sequences were clustered in operational taxonomic units (OTUs) at 97% similarity level using Uparse implemented within Usearch (Edgar, 2013). Taxonomic identities were assigned to the most abundant sequences of each OTU using Blastn v.2.5.0 searches against the UNITE 8.2 (Kõljalg *et al*., 2013) and National Center for Biotechnology Information (NCBI) databases. We assigned OTUs to taxonomic levels based on sequence identity thresholds: 90% for genus, 85% for family, 80% for order and 75% for class. Nontarget sequences, sequences with no hits as well as those with a coverage < 70% and an *e*‐value above the threshold 10^−40^, were disregarded. The *e*‐value threshold was set to 10^−40^ to avoid nonfungal organisms (Tedersoo *et al*., 2014). As the 10^−40^ threshold discriminated against some fungal groups with particularly short ITS reads (e.g. members of the Archaeorhizomycetes), the data were checked manually and those OTUs with similarity and coverage > 80% and the best hit assignments to a UNITE Species Hypothesis (SH) were retained as well. Fungal OTUs with a similarity > 90% to known SH were, based on their genus classification, assigned to a putative functional group according to their primary lifestyle in FungalTraits database (Polme *et al*., 2021) and characterized as saprotrophs, plant pathogens, ECM fungi, root endophytes or others.

### Statistical analysis

The same elevation may indicate varying positions on different mountain slopes. For instance, 300 m in Norway might be close to the midrange, whereas in the French Alps, it is near the lowland. Therefore, to enable the comparison of elevation gradients from diverse locations, we normalized elevation within a bounded domain from 0 to 1. On this scale, 0 represents sea level, and 1 is situated on the treeline. So, for instance, 0.5 consistently refers to the midpoint between sea level and the treeline (Table S1). We plotted climatic factors against elevation, normalized elevation and latitude to evaluate the effect of this normalization on the distributions of climatic factors (Fig. S1). Normalization did not alter distributions compared to raw elevation but reduced the bias of low‐elevation/low‐temperature points. This adjustment made the mean annual temperature (MAT) distribution along the elevational gradient more similar to the latitudinal one, in line with our study design objective to support meaningful comparisons between the two gradients. Further occurrences of ‘elevation’ in the Materials and Methods and Results sections of this article refer to this normalized elevation. All statistical analyses were performed in R v.3.6.3 (R Core Team, 2022) using the packages vegan (Oksanen, 2007) and dplyr (Wickham *et al*., 2023). Specifically, inext (Hsieh *et al*., 2016) was used for the OTUs richness estimation; lme4 (Bates *et al*., 2015), lmertest (Kuznetsova *et al*., 2017), mumin (Barton, 2009), mgcv (Wood, 2011), visreg (Breheny & Burchett, 2017) and viridis (Garnier *et al*., 2024) for the modeling of OTUs richness pattern; partr2 (Stoffel *et al*., 2024) to compute specific *R* ^2^ of generalized/linear mixed models (GLMM/LMM); hmsc (Tikhonov *et al*., 2020) for the Joint Species Distribution Modeling; and randomforest (Liaw & Wiener, 2001) and caret (Kuhn, 2008) for the evaluation of predictors of fungal OTUs richness.

#### OTUs richness estimation

Fungal diversity in this article refers to the number of OTUs, also known as OTUs richness. We used the observed number of OTUs and the Hill number, an estimation of the richness after normalization (Chao *et al*., 2014).

First, the observed OTUs richness (Sobs) corresponds to the number of OTUs present in each sample after elimination of singletons, that is a total of 19 513 fungal OTUs in the 159 samples studied. Specifically considering the main fungal guilds, 3058 OTUs were assigned to the ECM fungi, 5380 OTUs to the SAP fungi (including soil, litter, wood and unspecified SAP fungi) and 329 OTUs to the REND fungi.

Second, to take into account differences in sequencing depth between different samples, it is preferable to have a normalized estimation of the OTUs richness. We used the ‘estimateD ()’ function of inext (Hsieh *et al*., 2016) to obtain a standardized estimation of OTUs richness (Hill number *q* = 0) for the main fungal guilds as well as for the whole fungi. The normalization was done by sample coverage (SC), a measure of sample completeness done by extrapolation or interpolation of the rarefaction curve based on the OTUs observed in each sample after the removal of the singletons. We selected SC levels ranging from 0.99 to 0.90 in steps of 0.01. According to the inext instructions, when more than double the observed sample size was requested to estimate OTUs richness for a sample, this sample was removed (see the number of samples (*n*) used for each SC level in Table S2).

#### Models of OTUs richness pattern

We modeled OTUs richness along elevation and latitude using GLMM/LMM and generalized additive mixed models (GAMMs). For each fungal guild, models were fitted with the richness observed (Sobs) or the estimated richness (*q* = 0) with the 10 levels of sample coverage as response variables. In LMMs and GAMMs, OTUs richness values were square‐root transformed to normalize the distribution. We also used a negative binomial (NB) distribution with GLMMs (Table S2; Methods S1).

1. First, LMMs were designed with *elevation* and *latitude* together as fixed effects. *Location* and *plot* were included as random effects (Table S2). To address linear and nonlinear relationship, we fitted models with linear and unimodal relationships (second‐degree polynomial) of OTUs richness with elevation and latitude, resulting in four different combinations (Methods S1). To identify the best LMMs, we selected the model with the lowest Akaike information criterion (AIC) as the final model.
  Model 1 ← lmer(Richness ~ poly(Elevation,2) + poly(Latitude,2) + (1|Location) + (1|Plot))
  Model 2 ← lmer(Richness ~ poly(Elevation,2) + Latitude + (1|Location) + (1|Plot))
  Model 3 ← lmer(Richness ~ Elevation + poly(Latitude,2) + (1|Location) + (1|Plot))
  Model 4 ← lmer(Richness ~ Elevation + Latitude + (1|Location) + (1|Plot))
2. Second, to confirm the result of LMMs simplification GAMMs were designed in a similar way including *elevation* and *latitude* as fixed effects and *location* and *plot* as random effects.
3. Finally, since richness estimation can be treated as a count value, we examined NB distribution using GLMMs. Nevertheless, inext yielded noninteger values for richness, requiring us to round up the richness estimates. Additionally, with this distribution, we needed to center, using the ‘scale (x, center = TRUE, scale = TRUE)’ function, the variables *elevation* and *latitude* before fitting the models using the ‘glmer.nb ()’ function. Then, we identified the best models by following the same approach as the previous LMMs.

To account for the potential for elevational trends in species richness to change as a function of latitude and vice versa, we modeled interaction terms between elevation and latitude, following the same approach as for the previous LMMs, where interaction was fitted with nonquadratic and quadratic effects. For the GAMMs, we selected models with or without interaction based on the lowest AIC (Method S1; Table S3).

#### Predictors of fungal OTUs richness

The C : N ratio, phosphorus (P) and pH were used as soil predictors. The 19 bioclimatic predictors from the CHELSA database (Karger *et al*., 2017) were downloaded for the location of each of our samples (Table S1). Some of the climatic variables were highly correlated. Thus, bioclimatic predictors that had the largest variance inflation factor (VIF) were removed until there was no coefficient of correlation between two variables higher than 0.7 and a VIF of < 3 for each variable (indicating limited correlation of that predictor with other predictors). Results were the same when climatic variables were fitted to elevation or latitude. Based on the above preselection, six bioclimatic predictors were selected for analyses: MAT (Bio1), isothermality (Bio3), temperature seasonality (Bio4), precipitation seasonality (Bio15), precipitation of warmest quarter (Bio18) and precipitation of coldest quarter (Bio19).

To compare climatic shifts along elevational and latitudinal gradients, we modeled the absolute difference in the six selected bioclimatic predictors relative to changes in latitude and elevation. This approach allowed us to assess whether the absolute difference for each predictor between two points correlated similarly with changes in elevation or latitude (Fig. S2). In general, climatic differences followed similar trends between the two gradients, with MAT and ‘precipitation of the coldest quarter’ showing close agreement, though the latitudinal gradient has a slightly steeper slope. By contrast, ‘precipitation seasonality’ and ‘precipitation of the warmest quarter’ showed little change along elevation. Notably, normalized elevation has a distinct effect on isothermality.

**Fig. 2:**
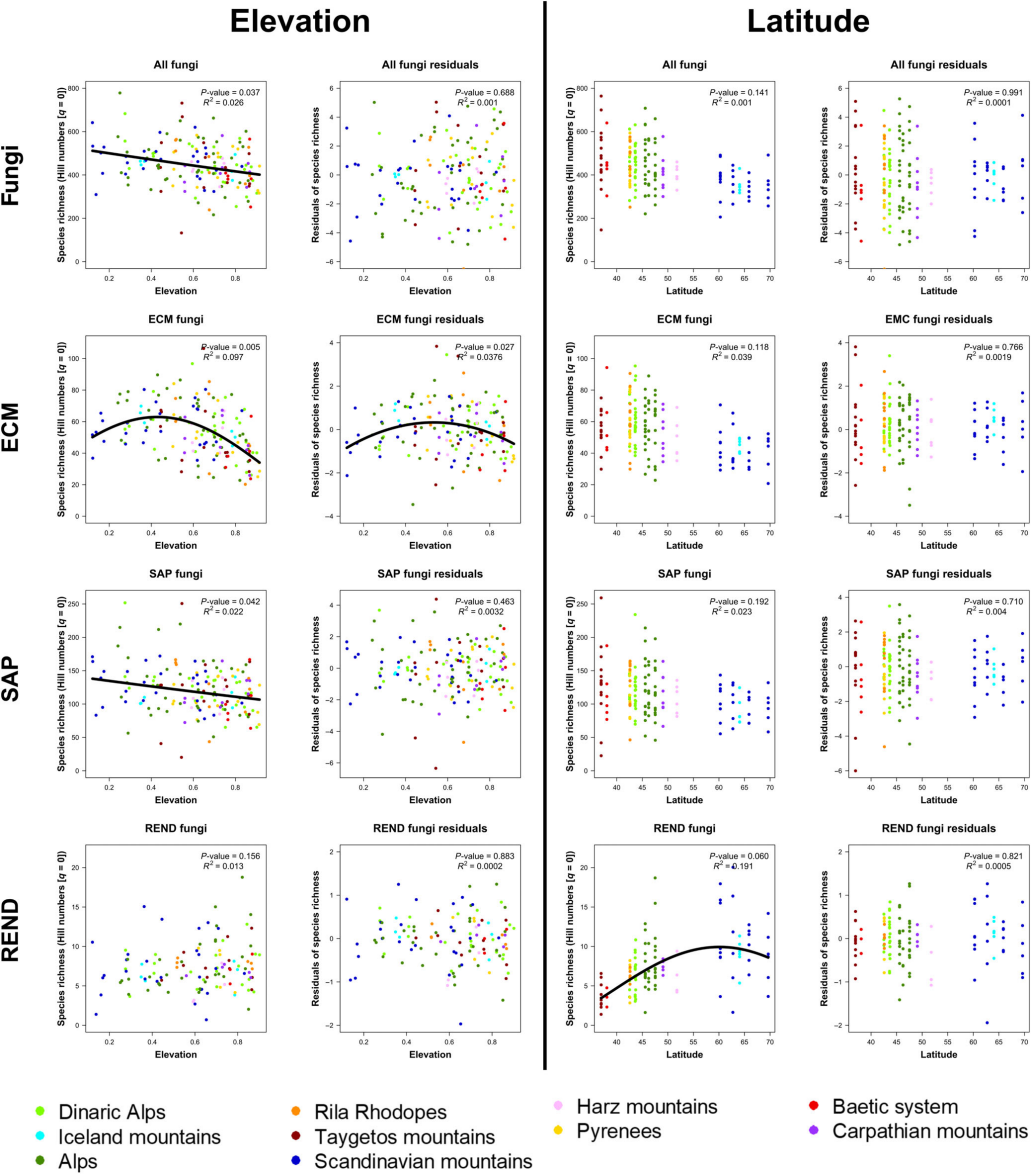
Fungal richness patterns along elevational and latitudinal gradients in Europe before and after accounting for soil and climatic variables. Fungal operational taxonomic units (OTUs) richness (left plots of each section) and residuals of fungal OTUs richness after accounting for climatic and soil predictors (right plots of each section) fitted along elevation (left section) and latitudinal (right section) gradients. The displayed plots represent a selection of the linear mixed model results, chosen based on a trade‐off between OTUs richness estimated with the highest sample coverage (SC) possible and a number of samples (*n*) used as close as possible to the maximum (159). For whole fungi (All), SC was set at 98% with *n* = 159. For ectomycorrhizal (ECM) fungi, SC was set at 99% with *n* = 157. For saprotrophic (SAP) fungi, SC was set at 97% with *n* = 159. For root endophytic (REND) fungi, SC was set at 94% with *n* = 121. The dots correspond to the different samples and are colored according to their mountain range of origin. Only significant (*P*‐value < 0.05) patterns are displayed with a full black line (see Supporting Information Figs S3–S6 for the full and detailed results of the fungal OTUs richness models).

To assess the importance of soil and climatic variables as responsible mechanisms in explaining diversity patterns, we fitted OTUs richness along elevation and latitude after accounting for the effect of environmental variables. To this purpose, we fitted OTUs richness with the six previously selected bioclimatic predictors and the soil variables using linear models. Subsequently, we used the residuals of these models as a response variable, in place of OTUs richness, in LMMs, following the previously described method and model selection (see ‘Models of OTUs richness pattern’ in the Materials and Methods section; Fig. 2).

Finally, the relative importance of climatic and soil variables in explaining fungal OTUs richness was calculated by a random forest analysis. Initially, a Random Forest model was trained using 500 trees. Following the training, the optimal number of predictor variables to consider at each split was determined using the ‘tuneRF ()’ function. Subsequently, the final model was rebuilt using the identified optimal number of variables (best.m), employing 1000 trees. To assess the stability of variable importance rankings, indicated by the mean %IncMSE, the process was repeated iteratively for 1000 iterations. The relationship between fungal OTUs richness and soil and climatic variables was examined using a mixed effects model, with Plot and Location included as random effects. Climatic and soil variables were standardized before analysis.

#### Hierarchical modeling of species communities

To model species‐level responses to elevation and latitude, we used joint species distribution modeling through the R package hmsc, a hierarchical model constructed in the generalized linear model (GLM) framework and using Bayesian inference (Ovaskainen & Abrego, 2020). Due to a low OTUs number and coverage of REND fungal groups, the occurrence probability was calculated for ECM and SAP fungi only. For this analysis, we applied a prevalence threshold and considered only OTUs that appeared in at least 10 samples (178 ECM fungi and 628 SAP fungi). We modeled the occurrence of OTUs with a binomial model with probit link function. As fixed explanatory variables, we included *elevation* and *latitude* at the second polynomial degrees, as well as log‐transformed *sequencing depth*, to account for the variation in sequencing depth among samples. *Location* and *Plot* were included as random effects. We fitted the models assuming the default priors and sampled the posterior distribution that ran four Markov chain Monte Carlo (MCMC) chains, each of which was run for 37 500 iterations, of which 12 500 were discarded as burn‐in. We thinned by 100 to obtain a total of 250 posterior samples per chain and 1000 posterior samples in total. We assessed MCMC convergence by measuring the potential scale reduction factor for the beta parameters (measuring the response to sample type) and considered the convergence satisfactory when they took values close to one. Then, we visually evaluated the response curves of random subsets of 100 ECM and 100 saprotroph OTUs to classify them into the different shapes that we found flat, unimodal hump‐shaped, unimodal U‐shaped, negative hump‐shaped, positive hump‐shaped, monotonic negative and monotonic positive. We further validated our approach by verifying whether the occurrence of a flat pattern (no significant positive or negative relationship with elevation and latitude at 90% posterior statistical support) was consistent between the subsamples of 100 OTUs and the full dataset of OTUs that appeared in at least 10 samples.

## Results

### Fungal OTUs richness distribution patterns

The combination of 17 elevation gradients from a single geographic region (Europe) enabled the detection of diversity patterns along elevation and latitude. We observed a significant decrease in the overall richness of fungal OTUs with increasing elevation. However, the results varied between fungal guilds. Specifically, SAP fungi showed a decreasing trend, whereas ECM fungi showed a richness peak at mid‐elevation. Interestingly, REND did not show any clear trend (Fig. 2). Regarding latitude, while we found no significant trends in overall, ECM and SAP fungal richness, REND fungi showed a trend opposite to the commonly reported gradient, namely a richness increase toward high latitude. These results held across different methods and levels of sample coverage (Figs S3–S6). We did not find any significant indications for the interaction effect between elevation and latitude on fungal richness (Table S3) and GAMMs, including interaction terms between elevation and latitude, had lower AIC than GAMMs with additive terms.

### Environmental predictors of fungal OTUs richness variation

**Fig. 3:**
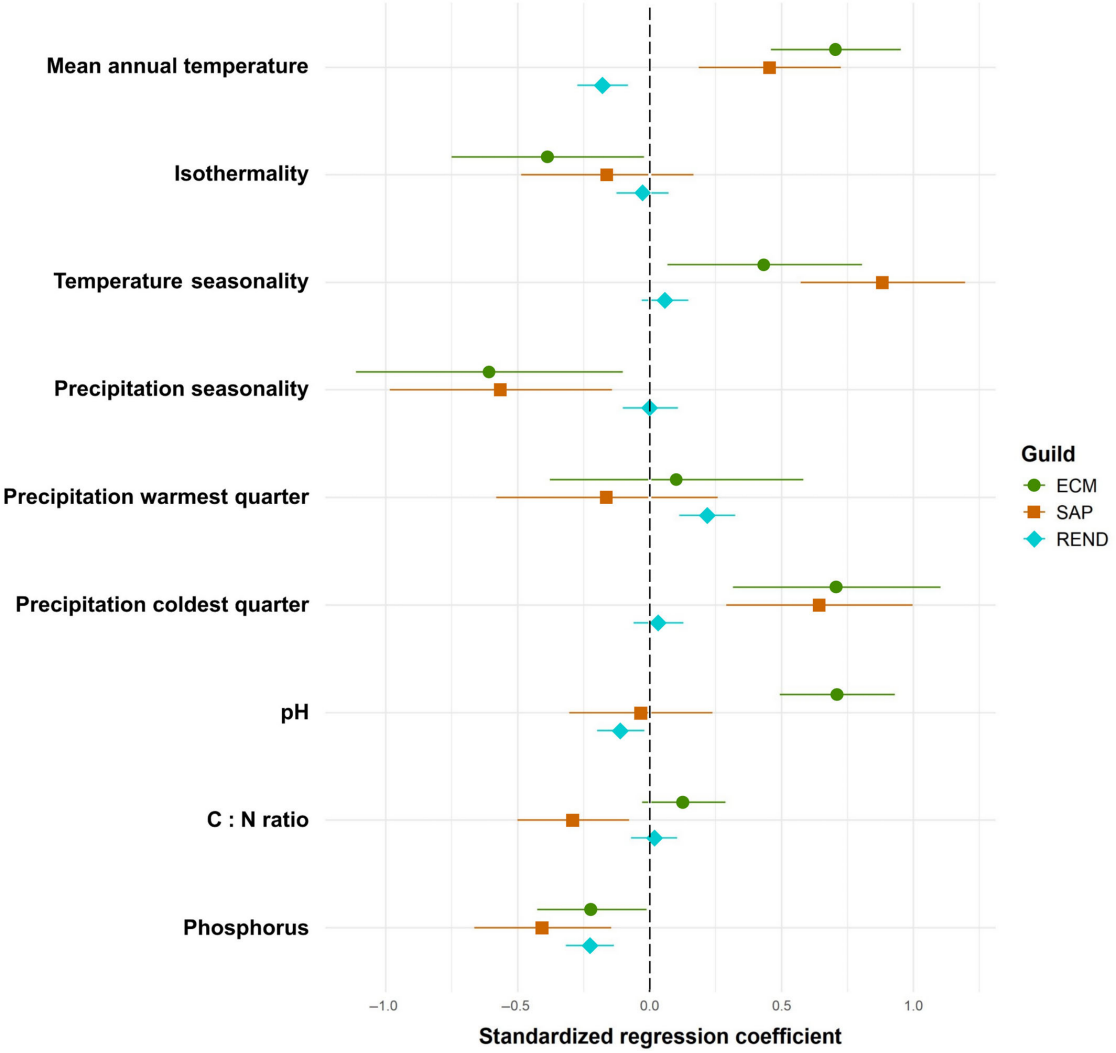
Direction and strength of the relationship between fungal species richness and climatic and soil variables. Forest plot representing, for each fungal guild (ectomycorrhizal (ECM) fungi in green, saprotrophic (SAP) fungi in brown and endophytic (REND) fungi in cyan), the standardised regression coefficient (95% confidence interval) of operational taxonomic unit (OTUs) richness – estimated with the highest sample coverage possible and a number of samples (*n*) used as close as possible to the maximum (159) – fitted with the climatic and soil variables. A bar across the dashed vertical line represents an absence of significant relationship, whereas a bar to the left or right indicates a negative and positive relationship, respectively.

We found that fungal richness is predicted by different factors across the three ecological guilds. The random forest models revealed strong effects of climatic seasonality for both temperature and precipitation on OTUs richness of ECM and SAP fungi. On the other hand, OTUs richness of REND fungi was rather affected by climate itself, mainly by MAT (Fig. S7). Interestingly, while ECM and SAP OTUs richness exhibited different patterns along studied gradients, they responded similar to climatic variables (Fig. 3). They were positively correlated to temperature seasonality, precipitation of coldest quarter and MAT and negatively correlated to precipitation seasonality. REND OTUs richness rather responds positively to precipitation of the warmest quarter and negatively to MAT. Among the soil characteristics, pH had a high impact on ECM and REND fungal richness, whereas OTUs richness of SAP fungi was also affected by phosphorus (P) (Figs S7, S8), even though this last result should be treated with caution because the P‐Olsen method may underperform in acidic soils. Although pH was the most prominent driver of ECM fungal richness, and a pH of 6 corresponded to the peak of OTUs richness, it did not change along elevation (Fig. S9). This suggests an independent role of pH on ECM fungal OTUs richness from elevation and climate characteristics. Importantly, the analyzed environmental factors were able to explain changes in OTUs richness along altitudinal and latitudinal gradients in case of whole fungi, SAP and REND, whereas hump‐shaped pattern of ECM fungal richness along elevational gradient remained significant even after accounting for the effects of climatic and soil characteristics (Fig. 2).

### Fungal OTUs occurrence patterns

**Fig. 4:**
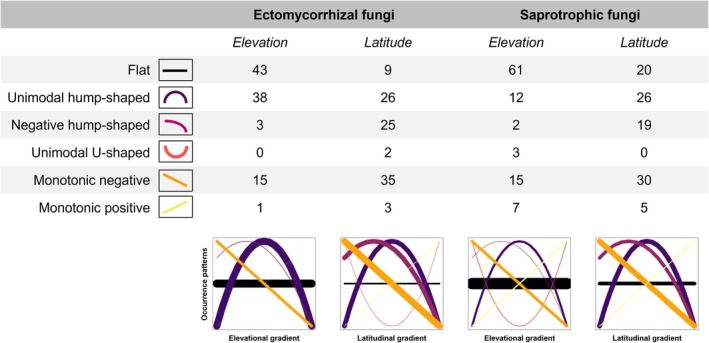
Joint species distribution modeling of species‐level responses to elevation and latitude. The table indicates the percentage of occurrence patterns for ectomycorrhizal and saprotrophic fungal operational taxonomic units, while the graphs illustrate these patterns, with line thickness proportional to their representativeness.

Almost 40% of tested ECM fungal OTUs displayed a unimodal hump‐shaped pattern of distribution along elevation, whereas monotonic positive or negative patterns were detected for < 15% of ECM fungal OTUs (Fig. 4). This indicates that high ECM fungal richness at mid‐elevations originates from specific occurrence of many OTUs in mid‐elevation sites, rather than overlapping ranges of OTUs from high and low elevations. Compared to ECM fungi, fewer SAP fungal OTUs (< 40% of tested OTUs) displayed a consistent shift along the elevation gradient, which corresponds to a weaker correlation between SAP fungal OTUs richness and elevation.

The prevailing pattern of fungal OTUs distribution along the latitudinal gradient corresponds to the observed weak negative correlations between ECM and SAP fungal OTUs richness and latitude. Specifically, 60% of tested ECM and 50% of tested SAP fungal OTUs displayed decreasing occurrence probability along latitude. Hump‐shaped distribution along the latitudinal gradient was identified for approximately the same proportion of ECM and SAP fungi, corresponding to 25% of tested OTUs.

## Discussion

While well documented in macroorganisms, elevation and latitude gradients remain less explored in fungi and we have limited consensus about their universality, shape and environmental drivers. By combining 17 elevation gradients sampled and processed consistently within the same geographic region, this study contrasts with previous meta‐analysis (Tedersoo *et al*., 2012, 2014; Hendershot *et al*., 2017; Větrovský *et al*., 2019) by facilitating robust comparisons of gradients. This approach provides a framework to better understand fungal biogeography and addresses these fundamental gaps. Although we found an overall monotonic decline in fungal diversity with elevation, the elevational gradients differed markedly across fungal guilds. For instance, ECM fungal richness peaked at mid‐elevations, whereas the richness of SAP fungi – the most diverse group, showed a weak declining tendency. However, these patterns were not confirmed along a latitudinal gradient. Conversely, REND fungal richness increased with latitude but remained comparable across elevations (Fig. 2). The existence of these contrasting patterns aligns with the observation that the most important drivers of richness differ among fungal ecological groups. Nonetheless, we identified similar significant effects of various climate characteristics (Fig. 3 – MAT, temperature and precipitation seasonality and precipitation during coldest quarter) on ECM and SAP fungal diversity, that also substantially changed along the elevation gradient. Although climate is recognized as a key driver of fungal species distribution (Steidinger *et al*., 2019; Větrovský *et al*., 2019; Mikryukov *et al*., 2023), our results reveal a more nuanced perspective. The dissimilarity in ECM and SAP fungal diversity patterns between latitudinal and elevational gradients, as well as their differing responses along elevation despite both guilds responding similar to the same climatic factors, suggests that climate, especially MAT, alone does not fully explain the observed patterns. This seems particularly evident in ECM fungi in European mountains where the hump‐shaped distribution of species richness along the elevational gradient prevailed even after accounting for the effect of climate and soil chemistry (Fig. 2).

The hump‐shaped relationship between ECM fungal OTUs richness and elevation corresponds to the commonly observed patterns for animals and plants (Brown, 2001; Rahbek, 2005; Colwell *et al*., 2016; Bueno *et al*., 2021). This pattern has been previously reported for ECM fungi on various mountain slopes, such as mount Fuji in Japan (Miyamoto *et al*., 2014), central Veracruz in Mexico (Gómez‐Hernández *et al*., 2011), Gaoligong mountains in China (Luo *et al*., 2023) and neotropical and paleotropical mountains in Malaysian Borneo (Geml *et al*., 2022). However, other studies found an increase in ECM fungal richness, such as in Chichibu in Japan (Shigyo & Hirao, 2021), and in neotropical and paleotropical mountains in Argentina (Geml *et al*., 2022), whereas others reported a decrease, for example in Hyrcanian forests in Iran (Bahram *et al*., 2012). These studies provided mixed results, but their comparability remains unclear, largely due to the use of different methodologies. Our results, based on consistent and comprehensive fungal sampling and sequencing, strongly support the hump‐shaped relationship, which appears to be the prevailing pattern of ECM fungal species richness along elevational gradients.

The peak of ECM fungal OTUs richness at mid‐elevations may be attributed to various processes. Traditionally, high species richness in mid‐elevations has been explained by the so‐called ‘mid‐domain effect’ (MDE). The MDE postulates that the hump‐shape gradient arises from geometric constraints, where the random placement of species ranges within bounded domain results in their overlap at the domain's center, leading to a higher number of observed species in the central region (Colwell *et al*., 2004). However, by modeling the probability of occurrence of ECM fungal OTUs, we found that the hump‐shaped richness pattern along the elevation gradient resulted from a higher likelihood of ECM fungal OTUs occurring at mid‐elevation sites. This pattern reflects a preference for mid‐elevation environmental conditions, as previously documented by Geml *et al*. (2017) rather than supporting the MDE hypothesis as suggested by Miyamoto *et al*. (2014), as relatively few fungi showed probabilities of occurrence that either consistently increased or decreased with elevation (Fig. 4). Moreover, evidence suggesting that the hump‐shaped pattern of ECM fungal richness does not originate from the MDE is also supported by diversity patterns of other fungal ecological groups along the studied elevational gradients. If the hump‐shaped species richness pattern along the elevational gradient were solely driven by geometric constraints, we would expect similar patterns across other fungal ecological groups. However, ECM fungi were the only group exhibiting a mid‐elevation peak in richness, indicating that additional, nonstochastic drivers are likely influencing this pattern.

Higher ECM plant diversity at mid‐elevation could contribute to ECM fungal species richness peak. However, the ECM tree diversity in our sampling sites was highly homogenous with no more than two dominant species per site, which cannot constitute a sufficient explanation. As obligate symbionts of various ECM plants, ECM fungi fully rely on their host plants as the only source of energy‐rich organic compounds (Smith & Read, 2008). The lower productivity of forest vegetation in high elevations can therefore result in lower ECM fungal growth and biomass, which would subsequently lead to lower ECM fungal diversity, as expected based on the more‐individuals hypothesis (Storch *et al*., 2018). However, this hypothesis cannot explain low ECM fungal richness in low elevations, as ECM tree species dominated along all studied elevational gradients. Therefore, we propose that lower ECM fungal diversity in low elevations is instead driven by the specificity of local environmental conditions, as low‐elevation forests are more prone to current and historical natural or anthropogenic disturbances (Stritih *et al*., 2021). For instance, low‐elevation forests are more susceptible to land use changes, intensive forestry and are more sensitive to climate change and high fire severity (Davis *et al*., 2019). The detrimental effects of land use on the species richness of plants and animals, resulting in hump‐shaped patterns in their diversity along the elevation gradient, have previously been documented on Mount Kilimanjaro (Peters *et al*., 2019). Considering that ECM fungal diversity is negatively affected by high land use intensity (Sterkenburg *et al*., 2019; Correia *et al*., 2021), we suggest that the hump‐shaped pattern of ECM fungal richness along elevational gradient results from a combination of factors that extend beyond climatic drivers, such as low resource availability in high altitudes while historical land use has a detrimental effect in lowlands. Nevertheless, this hypothesis requires future research, particularly on the legacy of anthropogenic disturbances on forest and ECM fungal diversity, to be validated.

Previous global scale studies documented a higher richness of ECM fungal species in temperate and boreal regions, including the majority of Europe (Tedersoo *et al*., 2014; Mikryukov *et al*., 2023), likely due to the high dominance of ECM host plants in the vegetation of these regions (Soudzilovskaia *et al*., 2019). Although our study did not detect any structuring of ECM fungal species richness along a latitudinal gradient across Europe, in contrast to Tedersoo *et al*. (2012), this may be attributed to the shorter scale of our gradient. Nevertheless, our findings provide clear evidence that soils with pH *c*. 6 tend to host the highest ECM fungal diversity. This corresponds with a previous global scale study, which identified pH as a key driver of fungal species richness, with ECM richness peaking in neutral soils, at pH 6 (Tedersoo *et al*., 2014). In summary, forests at mid‐elevational sites on neutral soils in European mountains might show the highest ECM fungal diversity. Preserving ECM fungi represents a critical challenge for the future, given the essential role of mycorrhizal fungi, particularly ECM, as key contributors to carbon stock in soil and their potential to mitigate climate change (Hawkins *et al*., 2023).

The OTUs richness of SAP fungi showed a tendency to decrease monotonously along the elevational gradient, but this trend was weakly supported by the occurrence of individual OTUs, suggesting a higher variation in SAP fungal OTUs distribution. The relationship between SAP species richness and elevation has been explored to a limited extent so far, with contrasting findings. For instance, along elevation gradients in neotropical and paleotropical forests SAP richness did not exhibit consistent patterns (Geml *et al*., 2022). By contrast, no significant elevation trends have been observed in European mountains, such as the Swiss Alps (Adamczyk *et al*., 2019) or the Austrian Alps (Bhople *et al*., 2022), while in cool‐temperate montane forests of Japan, SAP richness decreased with elevation (Shigyo & Hirao, 2021). The decline in SAP fungal diversity along the elevation gradient mirrors the global latitudinal pattern suggested by Tedersoo *et al*. (2014), implying a potential role of climate as a key driver of SAP diversity. Our random forest analysis lends partial support to this idea by showing that temperature seasonality and precipitation seasonality were the two most crucial factors influencing SAP richness before pH, corresponding to a recent global scale study, which identified climate as an important driver of SAP fungal species richness on a global scale (Mikryukov *et al*., 2023). Although we observed only a subtle trend, or even the absence of a decline in OTUs richness along the latitudinal gradient in Europe, diverging from the proposed global pattern, the individual occurrences of SAP fungal OTUs along our European latitudinal gradient revealed that a substantial portion of individual SAP fungal OTUs still tends to decline along latitude.

The REND fungi were the only ecological group that increased in richness along the studied latitudinal gradient. The ecological group of REND includes fungal species with versatile ecologies, all of which share the ability to asymptomatically colonize roots of a broad spectrum of plant species (Jumpponen & Trappe, 1998; Lukešová *et al*., 2015). They can play a significant role in carbon sequestration (Clemmensen *et al*., 2013, 2015) and the structuring of microbial communities (Netherway *et al*., 2024) in some of the forest soils. Ericoid mycorrhizal fungi (Chambers *et al*., 2008; Vohník *et al*., 2013) and dark‐septate endophytes (Jumpponen & Trappe, 1998) are among the most common types of REND fungi and they are frequently found in the roots of ericoid plants (Leopold, 2016; Kohout, 2017; Vohník, 2020). Unlike obligate mycorrhizal symbionts, such as arbuscular and ECM fungi, ericoid mycorrhizal fungi as well as other REND fungi possess large spectra of extracellular enzymes enabling them to switch into at least partial SAP lifestyle (Martino *et al*., 2018; Perotto *et al*., 2018). Due to the rather lower tolerance of some SAP fungal species to low temperatures compared to other fungal ecological groups (Qin *et al*., 2023) and the high abundance of ericoid vegetation in high latitudes (Bueno *et al*., 2017; Soudzilovskaia *et al*., 2019), REND fungi could better compete with SAP fungi in this niche, leading toward REND fungal richness peaks in high latitudes. We hypothesize that the less pronounced shift in REND fungal OTUs richness along elevation compared to the latitudinal gradient likely originates from the lower abundance of ericoid plants in high altitudes compared to high latitudes. However, the importance of various environmental characteristics influencing the species richness and distribution of REND fungi remains to be addressed in future studies.

We have highlighted the variation in fungal diversity along altitudinal and latitudinal gradients, based on a comprehensive survey covering much of the European continent. The majority of the observed patterns of fungal richness can be explained as a response of fungal richness to changes in environmental conditions (climatic and edaphic) as a function of latitude and altitude, with the exception of the relationship between elevation and ECM fungal richness, which remained hump‐shaped even after accounting for the effects of environmental conditions on species richness. This suggests that factors driving ECM fungal species richness along elevation are not solely related to climate or soil. We speculate that human activities, associated with intensified forest management, might be responsible for the decline in ECM fungal richness at low elevations. Our results pave the way toward a more comprehensive understanding of fungal diversity patterns, their generalities and idiosyncrasies, which could serve as groundwork for advancing the field of microbial biogeography research.

## Competing interests

None declared.

## Authors contributions

PK coordinated this project. FB, TM and PK designed the study. TM, IO, A Moravcová, CA, DB, SB, VB, SAH, ZH, HK, JL, CL, LM, DKM, NN, ET, VT, TV and PK participated in the fieldwork. TM and PK made the data curation. FB, TM and IO analyzed the data. FB wrote the original draft. TM, IO, A Machac, PB and PK reviewed the first draft. All the authors reviewed and contributed to the final version of the manuscript. FB and TM contributed equally to this work.

## Disclaimer

The New Phytologist Foundation remains neutral with regard to jurisdictional claims in maps and in any institutional affiliations.

## Supporting information


**Fig. S1** Climatic factor changes along elevation and latitude.
**Fig. S2** Difference of climate fitted with difference of elevation and latitude.
**Fig. S3** Whole fungal richness patterns along elevational and latitudinal gradients in Europe.
**Fig. S4** Ectomycorrhizal fungal richness patterns along elevational and latitudinal gradients in Europe.
**Fig. S5** Saprotrophic fungal richness patterns along elevational and latitudinal gradients in Europe.
**Fig. S6** Root endophytic fungal richness patterns along elevational and latitudinal gradients in Europe.
**Fig. S7** Relative importance of variables in explaining variation in fungal richness.
**Fig. S8** Relative importance of variables in explaining variation in fungal richness, all results.
**Fig. S9** Additional information about pH.
**Methods S1** Summary of the different modeling approaches and plots of residuals against fitted values and QQ plots for the representative datasets (FUNGI.98, ECM.99, SAP.97 and REND.94) used in LMM, GAMM and GLMM.NB.

**Table S1** Information and environmental parameters of the samples.

**Table S2** Summary of fungal species richness models estimated with different levels of sample coverage.

**Table S3** Summary of fungal species richness models estimated with different levels of sample coverage and using an interaction term between elevation and latitude.
Please note: Wiley is not responsible for the content or functionality of any Supporting Information supplied by the authors. Any queries (other than missing material) should be directed to the *New Phytologist* Central Office.

## Data Availability

Sequence data generated during the current study are deposited in SRA (BioProject PRJNA869039), https://www.ncbi.nlm.nih.gov/bioproject/?term=PRJNA869039.
