# Supplementary material for: Disentangling drivers behind fungal diversity gradients along altitude and latitude
Source: New Phytol. 2025 Feb 25;247(1):295–308. doi: 10.1111/nph.70012 (PMC12138170; doi:10.1111/nph.70012)
Supplement: Supplementary file 1 — Fig. S1 Climatic factor changes along elevation and latitude. Fig. S2 Difference of climate fitted with difference of elevation and latitude. Fig. S3 Whole fungal richness patterns along elevational and latitudinal gradients in Europe. Fig. S4 Ectomycorrhizal fungal richness patterns along elevational and latitudinal gradients in Europe. Fig. S5 Saprotrophic fungal richness patterns along elevational and latitudinal gradients in Europe. Fig. S6 Root endophytic fungal richness patterns along elevational and latitudinal gradients in Europe. Fig. S7 Relative importance of variables in explaining variation in fungal richness. Fig. S8 Relative importance of variables in explaining variation in fungal richness, all results. Fig. S9 Additional information about pH. Methods S1 Summary of the different modeling approaches and plots of residuals against fitted values and QQ plots for the representative datasets (FUNGI.98, ECM.99, SAP.97 and REND.94) used in LMM, GAMM and GLMM.NB. [file NPH-247-295-s001.pdf]

New Phytologist

***New Phytologist* Supporting Information**

Article title: Disentangling drivers behind fungal diversity gradients along altitude and latitude

Authors: Florian Barbi*,†, Tijana Martinović*, Iñaki Odriozola, Antonin Machac, Andrea Moravcová, Camelia Algora, Dalibor Ballian, Sebastian Barthold, Vendula Brabcová, Sandra Awokunle Hollá, Zander Human, Hojka Kraigher, Jelena Lazarević, Clementine Lepinay, Lenka Mészárošová, Daniel Kumazawa Morais, Nikolai Nikolov, Ella Thoen, Vojtěch Tláskal, Tomáš Větrovský, Petr Baldrian, Petr Kohout†

* These authors contributed equally to this work.
† Corresponding authors

Article acceptance date: 22 January 2025

The following Supporting Information is available for this article:

Fig. S1 Joint species distribution modelling of species-level responses to elevation and latitude

Fig. S2 Difference of climate fitted with difference of elevation and latitude

Fig. S3 Whole fungal richness patterns along elevational and latitudinal gradients in Europe

Fig. S4 Ectomycorrhizal (ECM) fungal richness patterns along elevational and latitudinal gradients in Europe

Fig. S5 Saprotrophic (SAP) fungal richness patterns along elevational and latitudinal gradients in Europe

Fig. S6 Root endophytic (REND) fungal richness patterns along elevational and latitudinal gradients in Europe

Fig. S7 Relative importance of variables in explaining variation in fungal richness

Fig. S8 Relative importance of variables in explaining variation in fungal richness, all results

Fig. S9 Additional information about pH

Table S1 Information and environmental parameters of the samples

Table S2 Summary of fungal species richness models estimated with different levels of sample coverage.

Table S3 Summary of fungal species richness models estimated with different levels of sample coverage and using interaction term between elevation and latitude

Methods S1 Summary of the different modelling approaches and plots of residuals

Fig. S1 *Climatic factor changes along elevation and latitude.* Linear regression between the six selected bioclimatic predictors and elevation, normalised elevation and latitude.

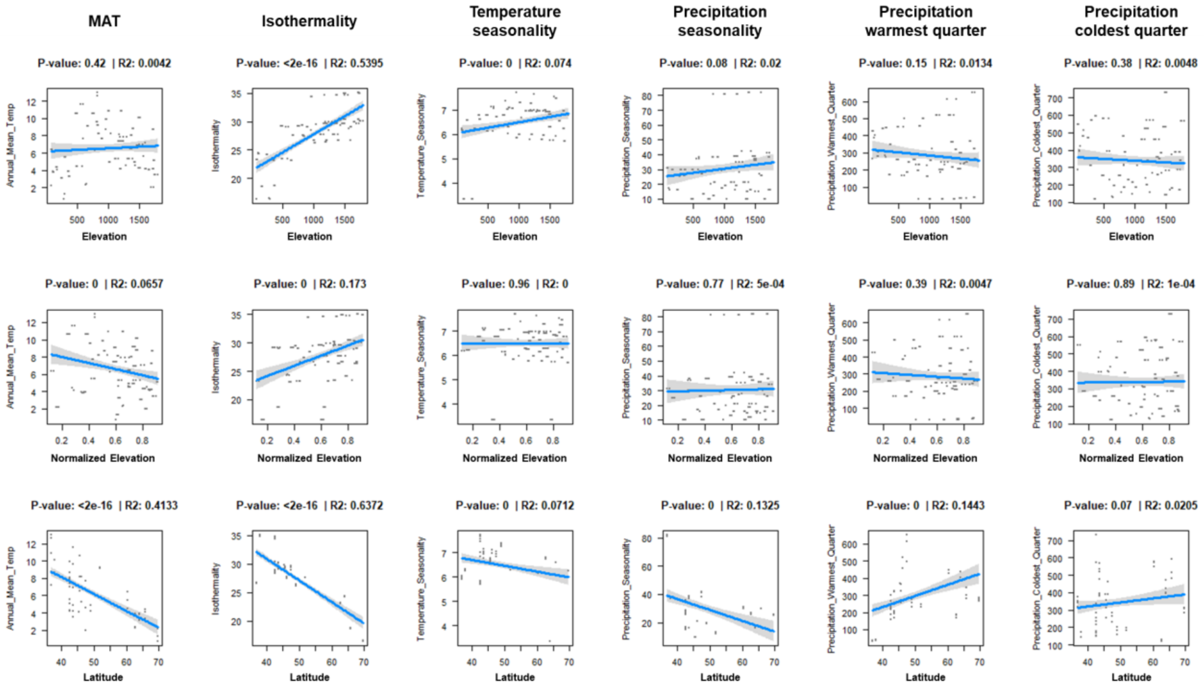

Fig. S2 *Difference of climate fitted with difference of elevation and latitude* Linear regression between the absolute difference in the six selected bioclimatic predictors relative to changes in latitude, elevation and normalised elevation.

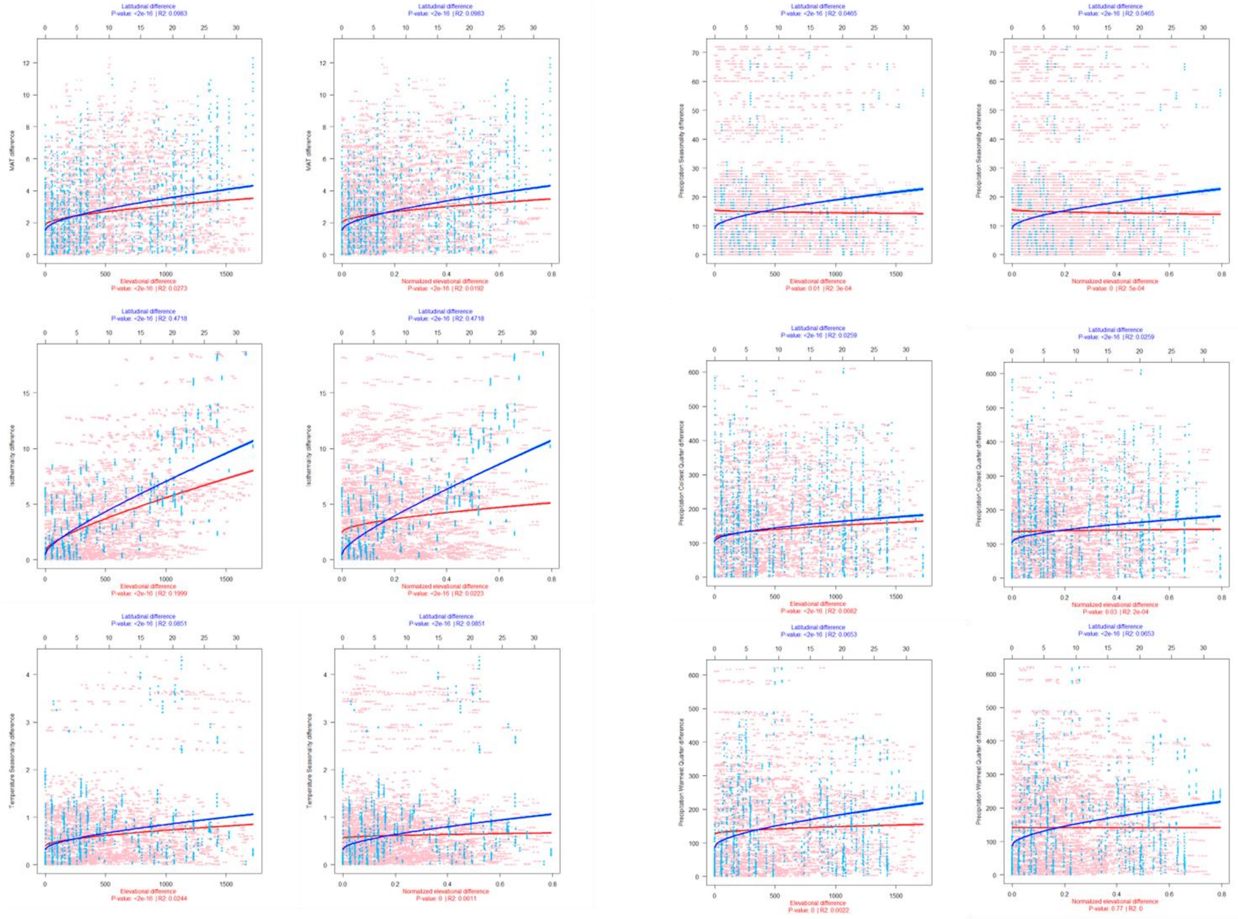

Fig. S3 *Whole fungal richness patterns along elevational and latitudinal gradients in Europe.* Patterns obtained from G/LMMs, GAMMs (Generalized / Linear Mixed Models and Generalized Additive Mixed Models using Gaussian distribution: square-root transformation of the estimated richness) and NB (G/LMMs using negative binomial distribution) for OTUs observed and richness estimated with different sample coverage, from 99% to 90%. (**A**) Models using normalised elevation and (**B**) models using raw elevation.

Supplementary Figures 3A – ALL fungi richness patterns – Normalized elevation

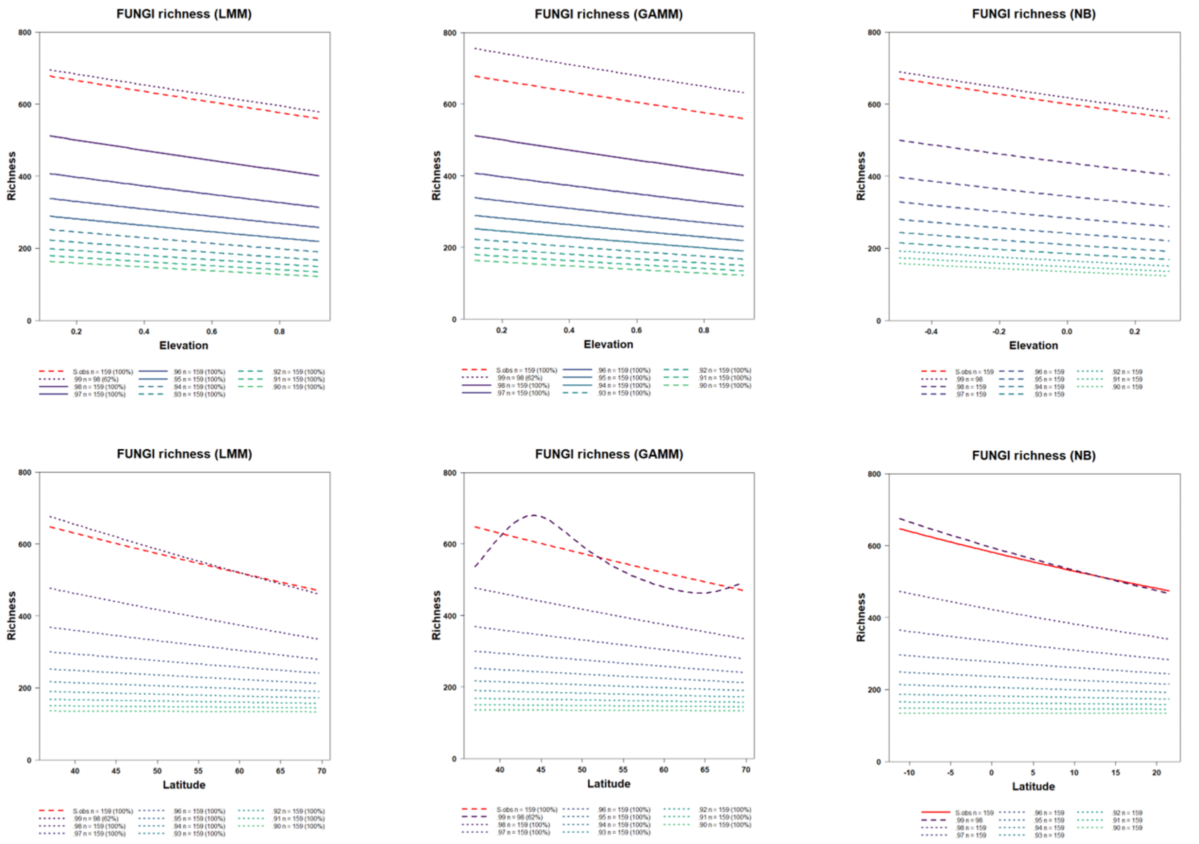

Supplementary Figures 3B – ALL fungi richness patterns – Raw elevation

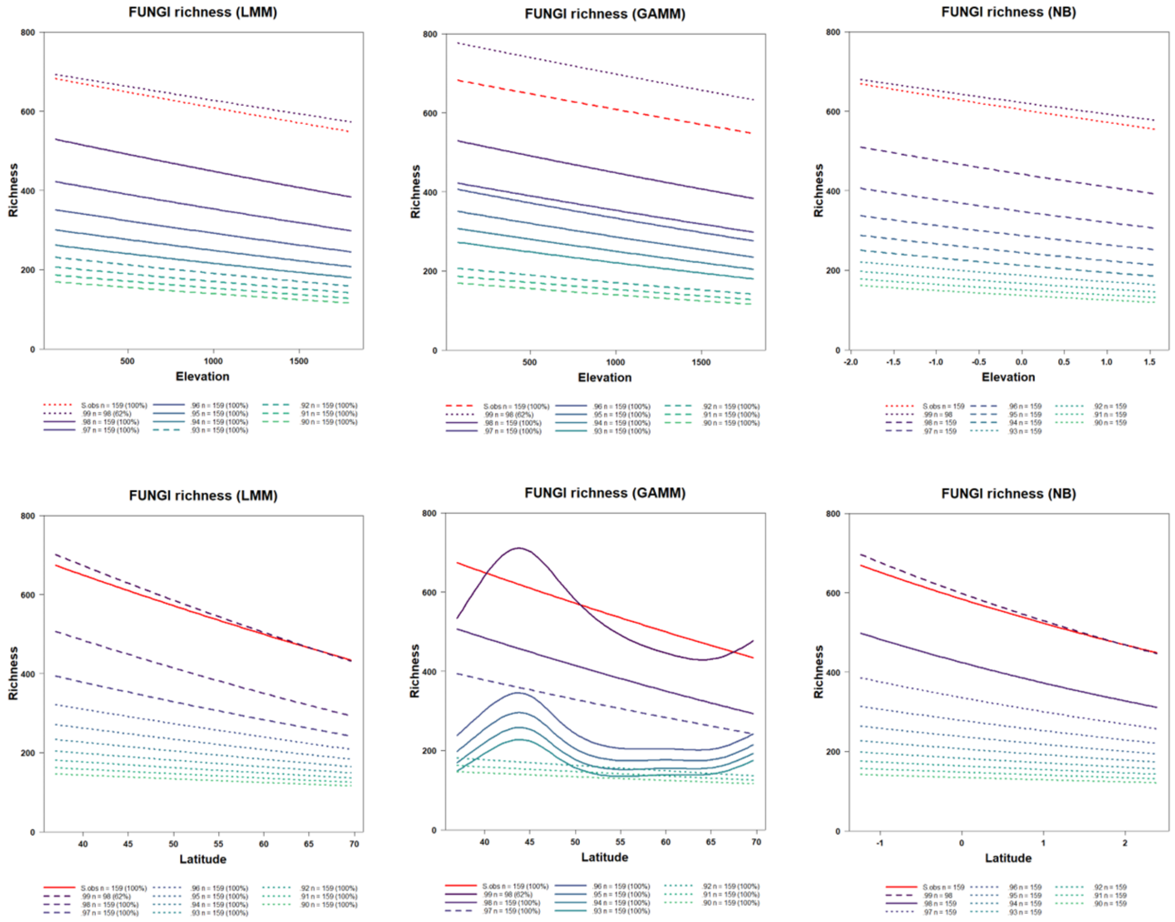

Fig. S4 *Ectomycorrhizal (ECM) fungal richness patterns along elevational and latitudinal gradients in Europe.* Patterns obtained from G/LMMs, GAMMs (Generalized / Linear Mixed Models and Generalized Additive Mixed Models using Gaussian distribution: square-root transformation of the estimated richness) and NB (G/LMMs using negative binomial distribution) for OTUs observed and richness estimated with different sample coverage, from 99% to 90%. (**A**) Models using normalised elevation and (**B**) models using raw elevation.

Supplementary Figures 4A – ECM fungi richness patterns – Normalized elevation

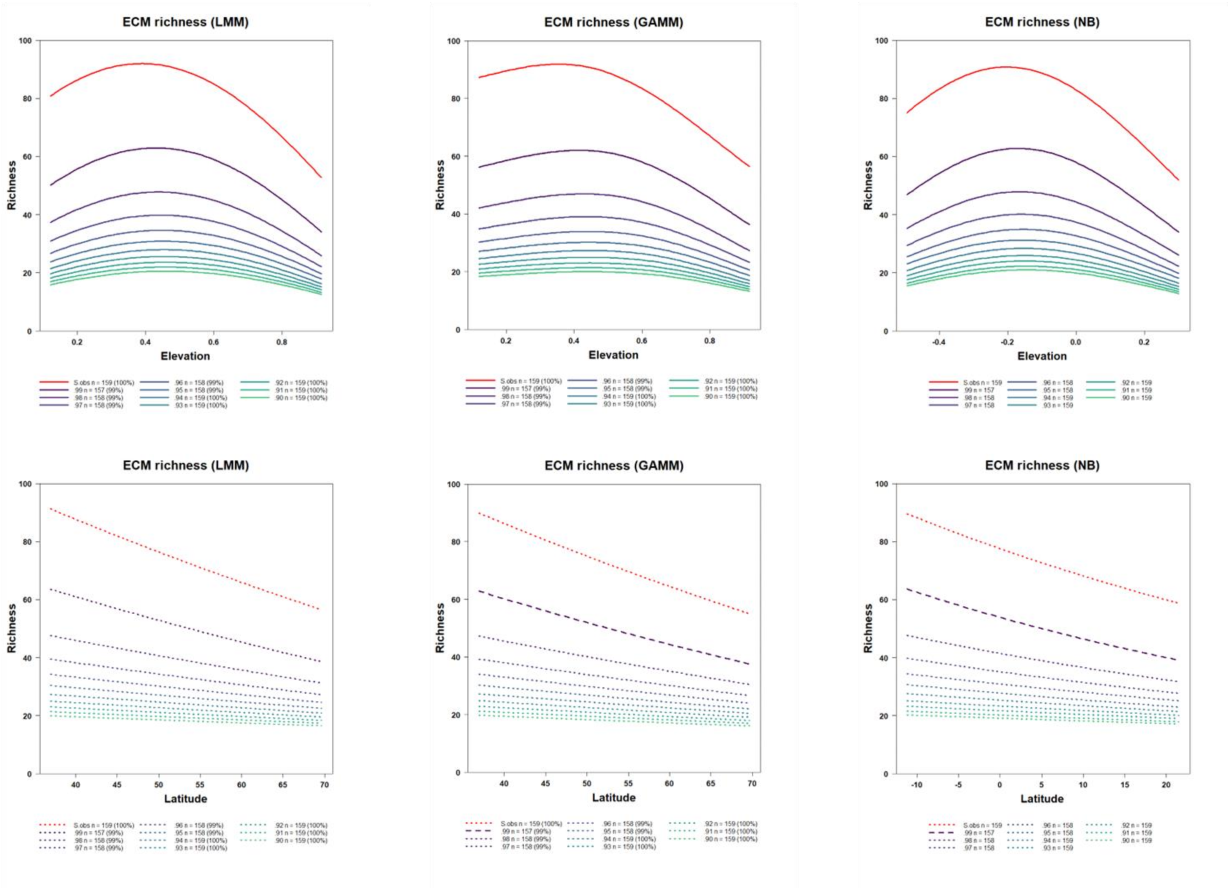

New Phytologist

Supplementary Figures 4B – ECM fungi richness patterns – Raw elevation

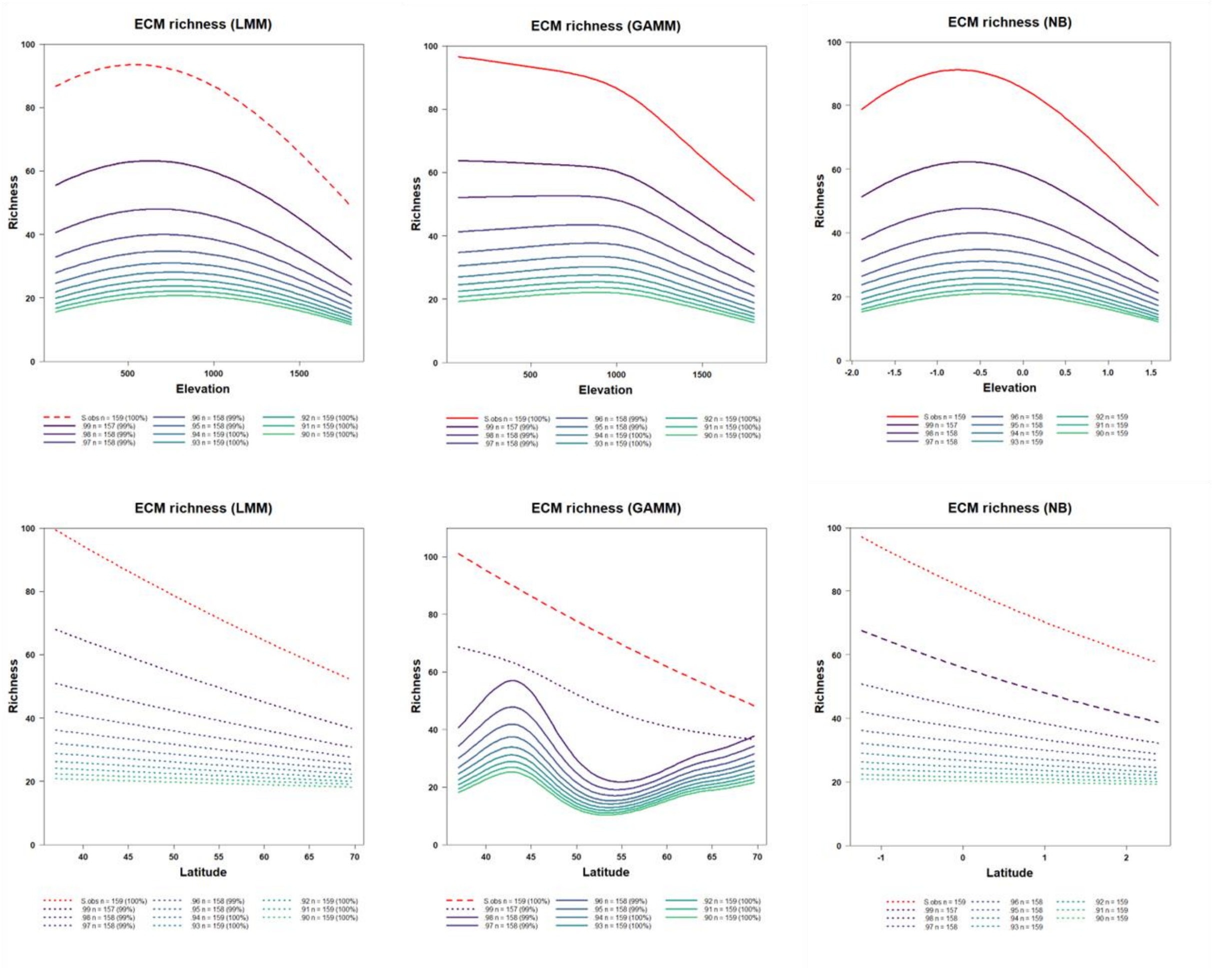

Fig. S5 *Saprotrophic (SAP) fungal richness patterns along elevational and latitudinal gradients in Europe.* Patterns obtained from G/LMMs, GAMMs (Generalized / Linear Mixed Models and Generalized Additive Mixed Models using Gaussian distribution: square-root transformation of the estimated richness) and NB (G/LMMs using negative binomial distribution) for OTUs observed and richness estimated with different sample coverage, from 99% to 90%. (**A**) Models using normalised elevation and (**B**) models using raw elevation.

Supplementary Figures 5A - SAP fungi richness patterns – Normalized elevation

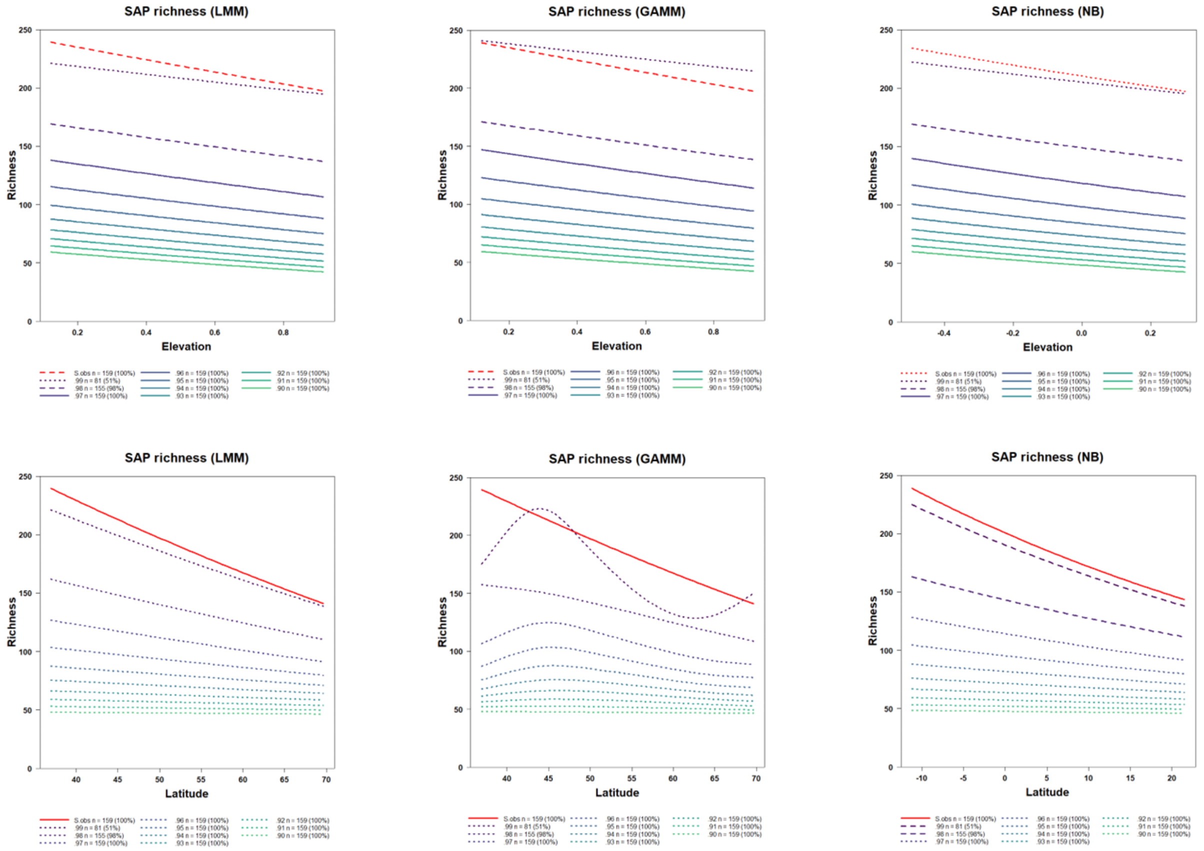

New Phytologist

Supplementary Figures 5B - SAP fungi richness patterns – Raw elevation

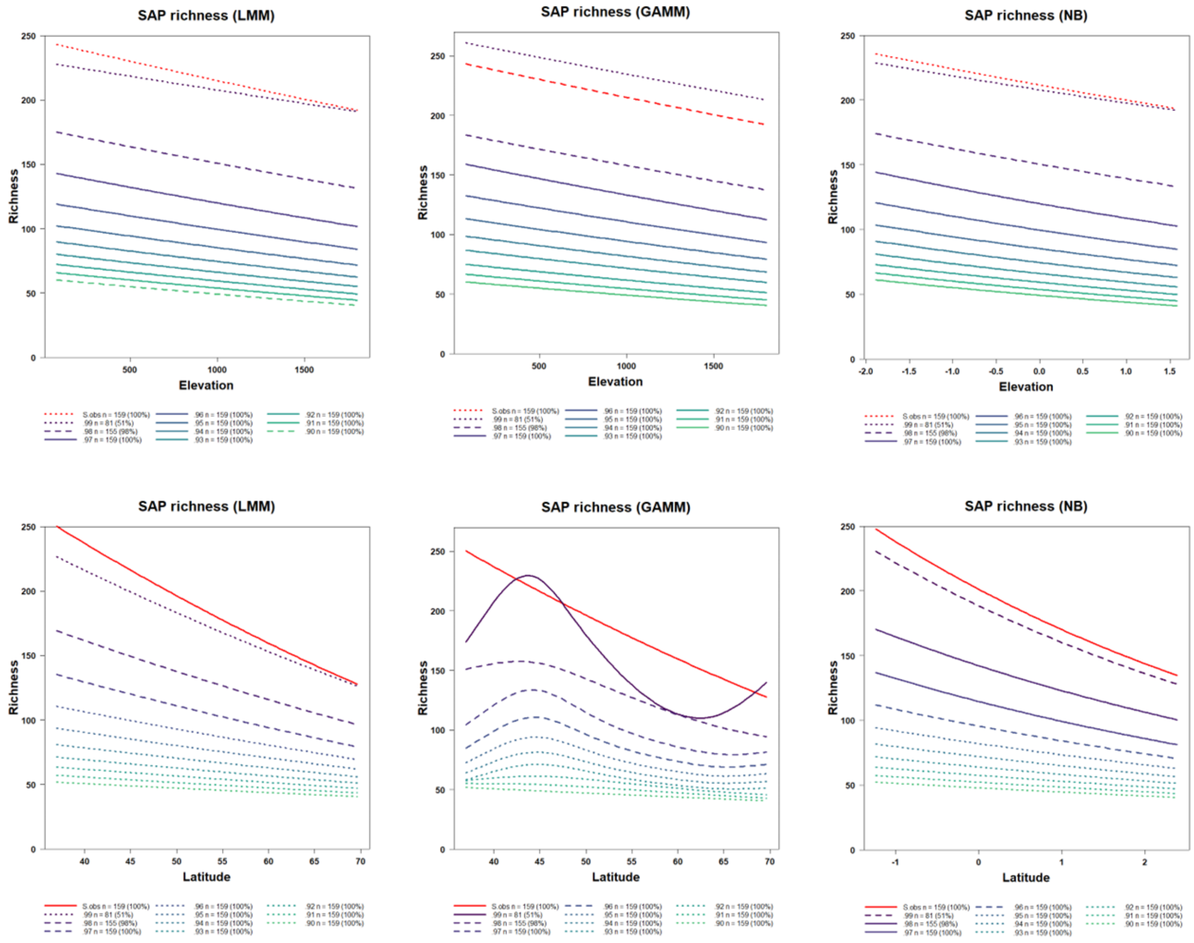

Fig. S6 *Root endophytic (REND) fungal richness patterns along elevational and latitudinal gradients in Europe.* Patterns obtained from G/LMMs, GAMMs (Generalized / Linear Mixed Models and Generalized Additive Mixed Models using Gaussian distribution: square-root transformation of the estimated richness) and NB (G/LMMs using negative binomial distribution) for OTUs observed and richness estimated with different sample coverage, from 99% to 90%. (**A**) Models using normalised elevation and (**B**) models using raw elevation.

Supplementary Figures 6A - REND fungi richness patterns – Normalized elevation

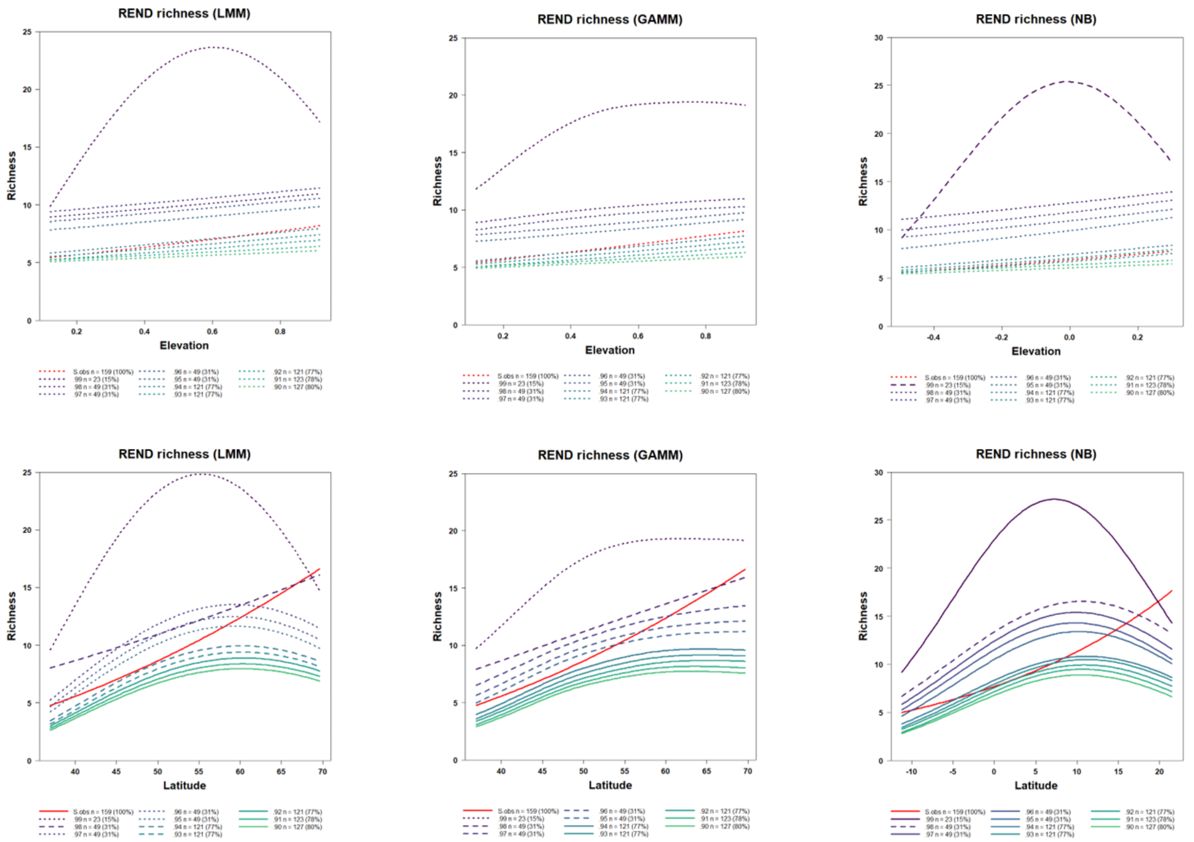

Supplementary Figures 6B - REND fungi richness patterns – Raw elevation

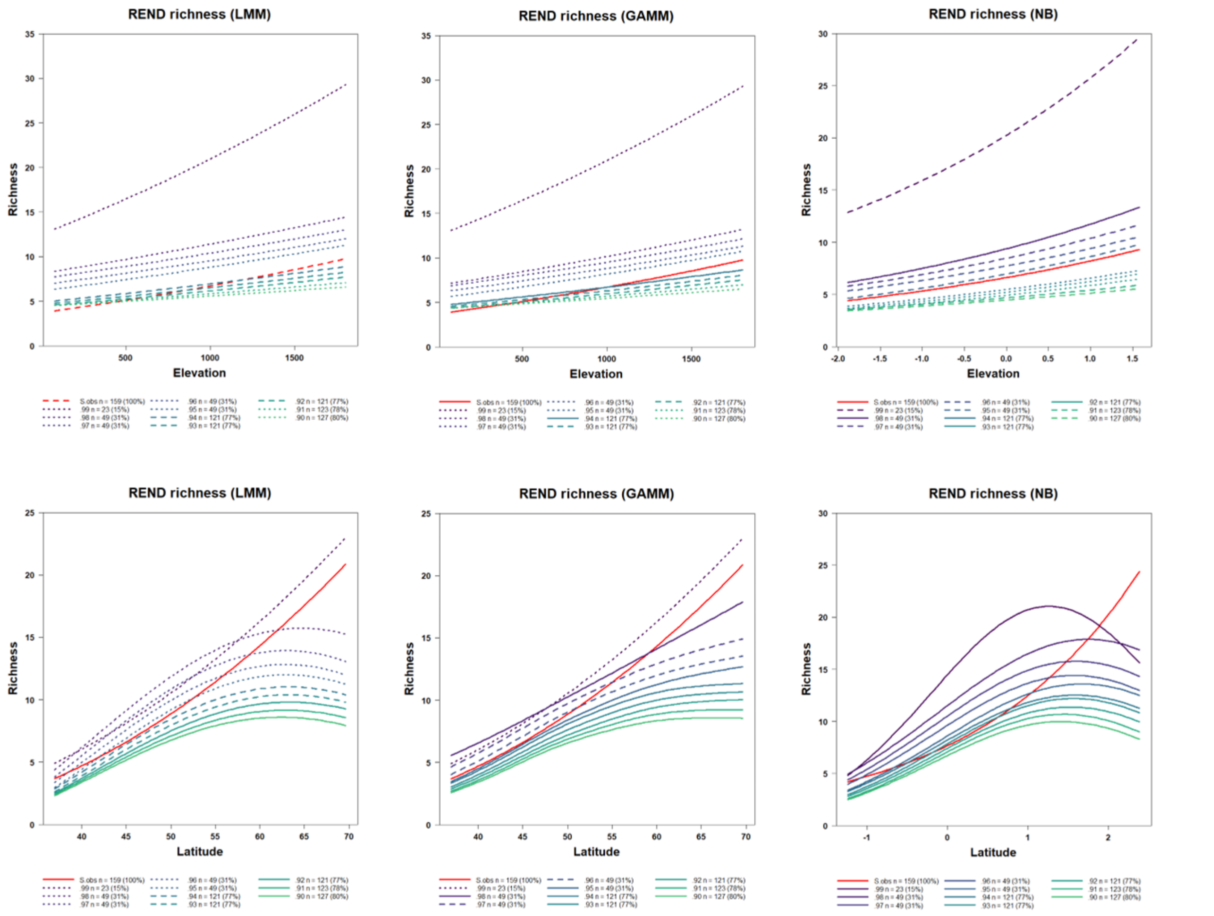

Fig. S7 *Relative importance of variables in explaining variation in fungal richness.* Random forest models were used to assess the importance of climatic (cyan) and soil (dark yellow) variables for predicting OTUs richness. Ranking of variables is based on the means %IncMSE of 1000 iterations of the model. We displayed a selection of the results, chosen based on a trade-off between OTUs richness estimated with the highest sample coverage (SC) possible and a number of samples (n) used as close as possible to the maximum (159). For ectomycorrhizal (ECM) fungi, SC was set at 99% with n=157. For saprotrophic (SAP) fungi, SC was set at 97% with n=159. For root endophytic (REND) fungi, SC was set at 94% with n=121. (see Supporting Information Fig. S7 for the full and detailed results)

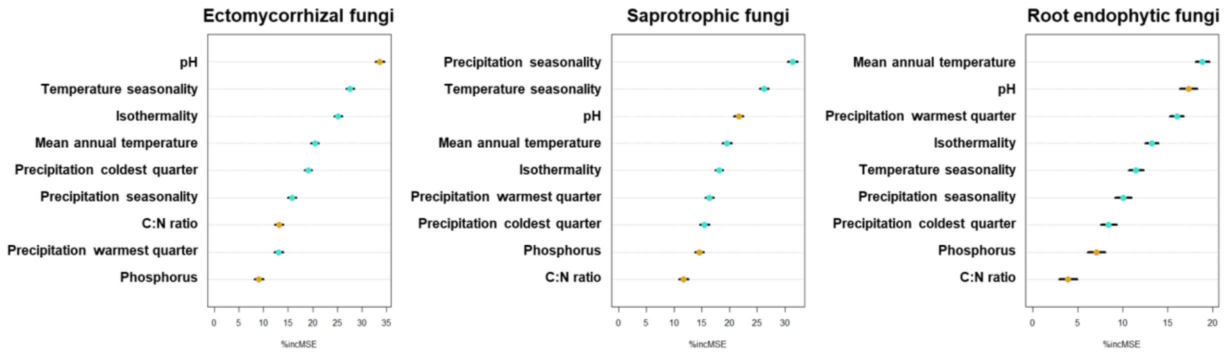

Fig. S8 *Relative importance of variables in explaining variation in fungal richness, all results.* Random forest models were used to assess the importance of climatic (cyan) and soil (dark yellow) variables for predicting the whole, ECM, SAP and REND fungal richness. Ranking of variables is based on the means %IncMSE of 1000 iterations of the model.

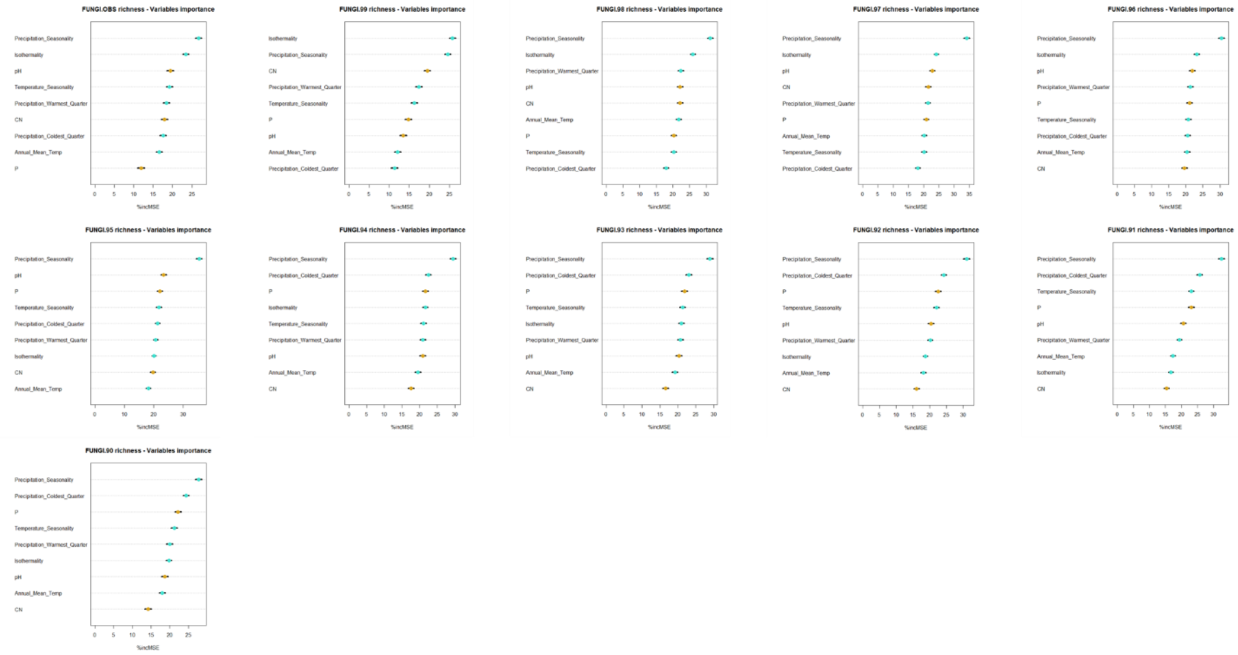

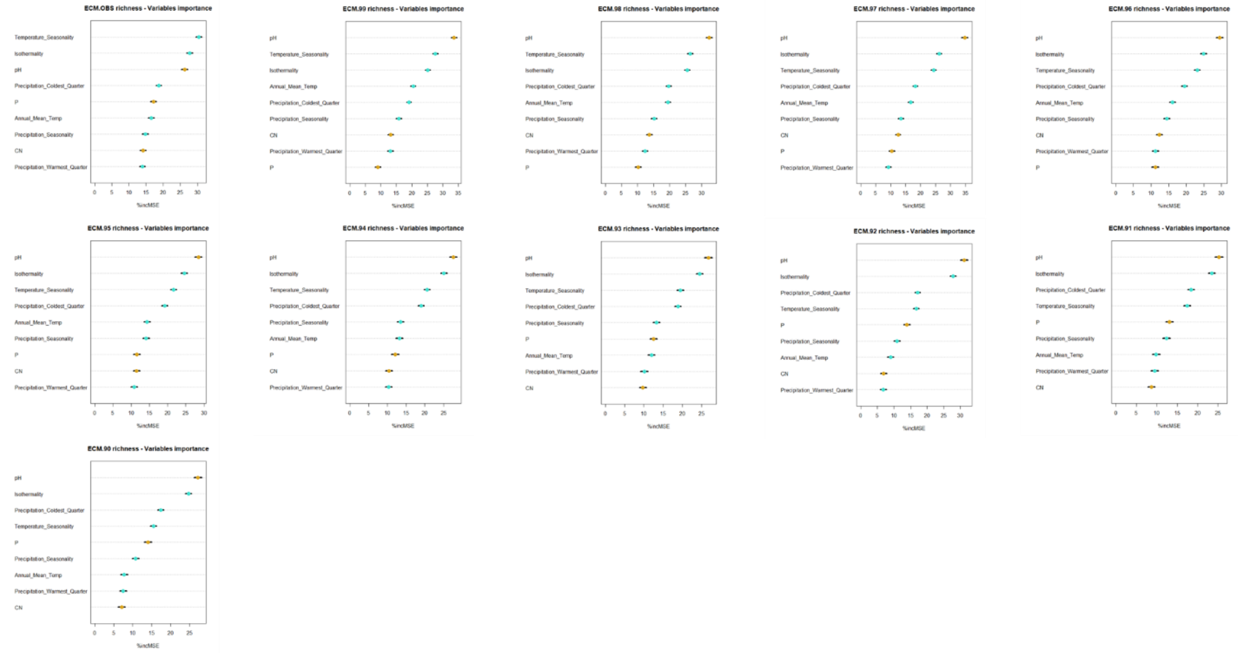

New Phytologist

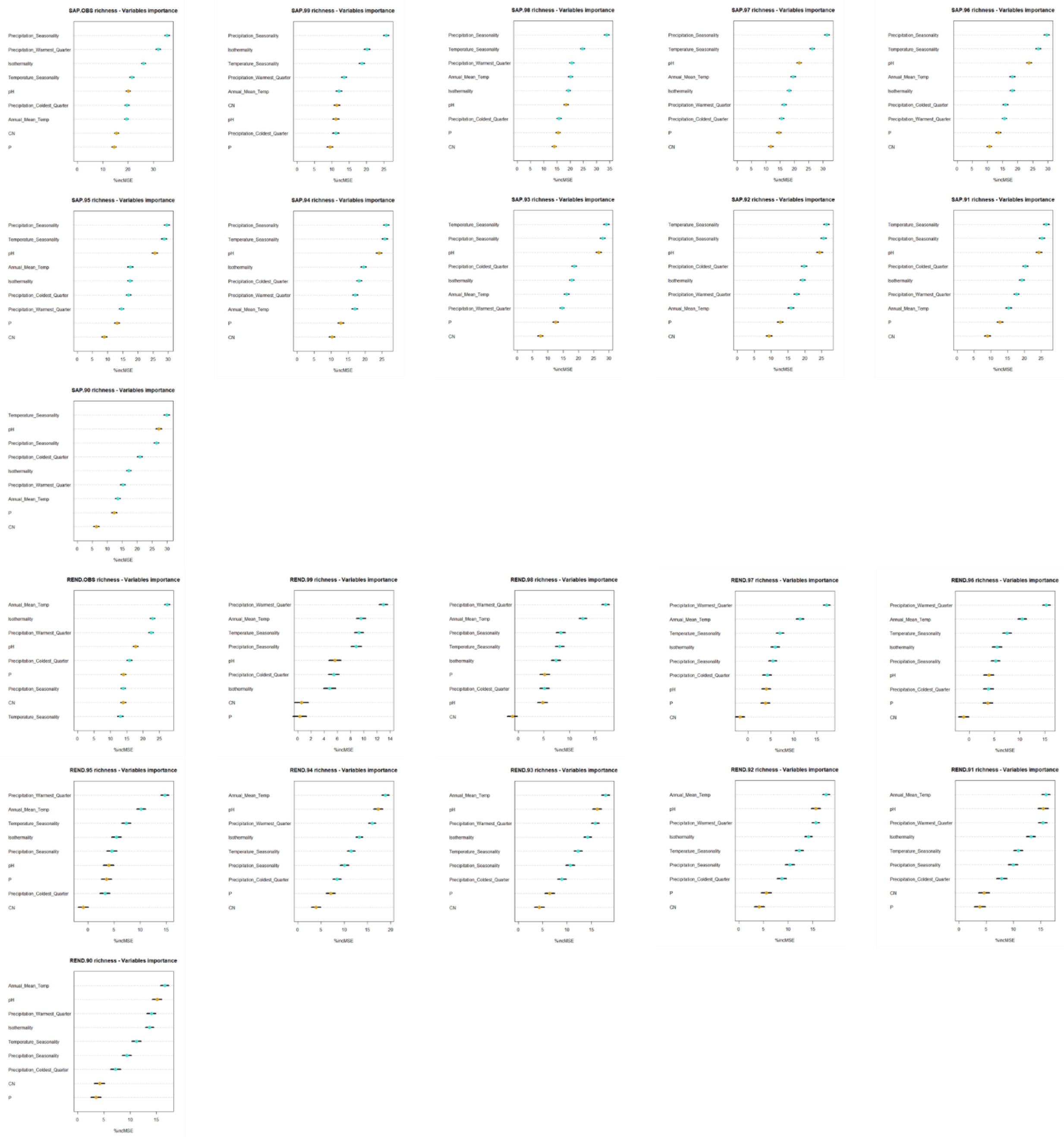

New Phytologist

Fig. S9 *Additional information about pH* A: Unimodal regression between ECM species richness and soil pH B: Non-significant relationship between soil pH and elevation

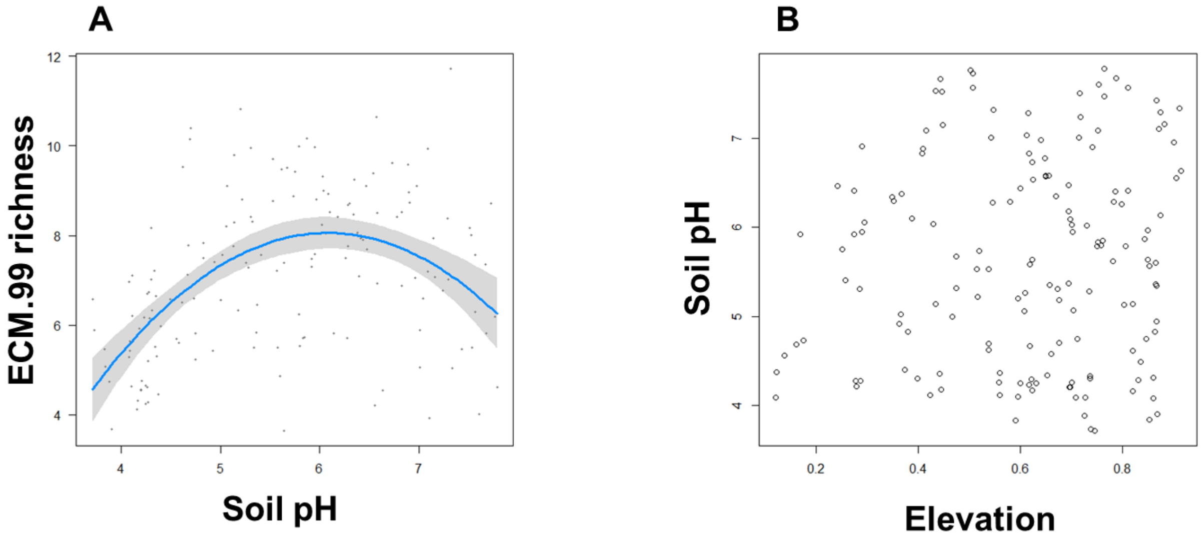

Table S1 Information and environmental parameters of the samples.

**Large table, see Excel file.*

Table S2 Summary of fungal species richness models estimated with different levels of sample coverage. The tables show for each model (i.e. LMMs, GAMMs (Linear Mixed Models and Generalized Additive Mixed Models using Gaussian distribution: square-root transformation of the estimated richness) and NB (GLMMs using negative binomial distribution): the model formulas, the number of sample (n) and the percentage it represented (n%) compared to the total number (159), the p-values (italic: $p<0.1$; bold $p<0.05$) and the R2 values for each fixed effect (Elevation and Latitude). The first and second tabs summarized the models using normalised elevation and raw elevation, respectively.

**Large table, see Excel file.*

Table S3 Summary of fungal species richness models estimated with different levels of sample coverage and using interaction term between elevation and latitude. The tables show for each model (i.e. LMMs, GAMMs (Linear Mixed Models and Generalized Additive Mixed Models using Gaussian distribution: square-root transformation of the estimated richness) and NB (GLMMs using negative binomial distribution): the model formulas, the number of sample (n) and the percentage it represented (n%) compared to the total number (159), the p-values and the R2 values (italic: $p<0.1$; bold $p<0.05$) for each fixed effect (Elevation and Latitude). The first and second tabs summarized the models using normalised elevation and raw elevation, respectively.

**Large table, see Excel file.*

Methods S1 Summary of the different modelling approaches and plots of residuals against fitted values and QQ plots for the representative datasets (FUNGI.98, ECM.99, SAP.97 and REND.94) used in LMM, GAMM and GLMM.NB.

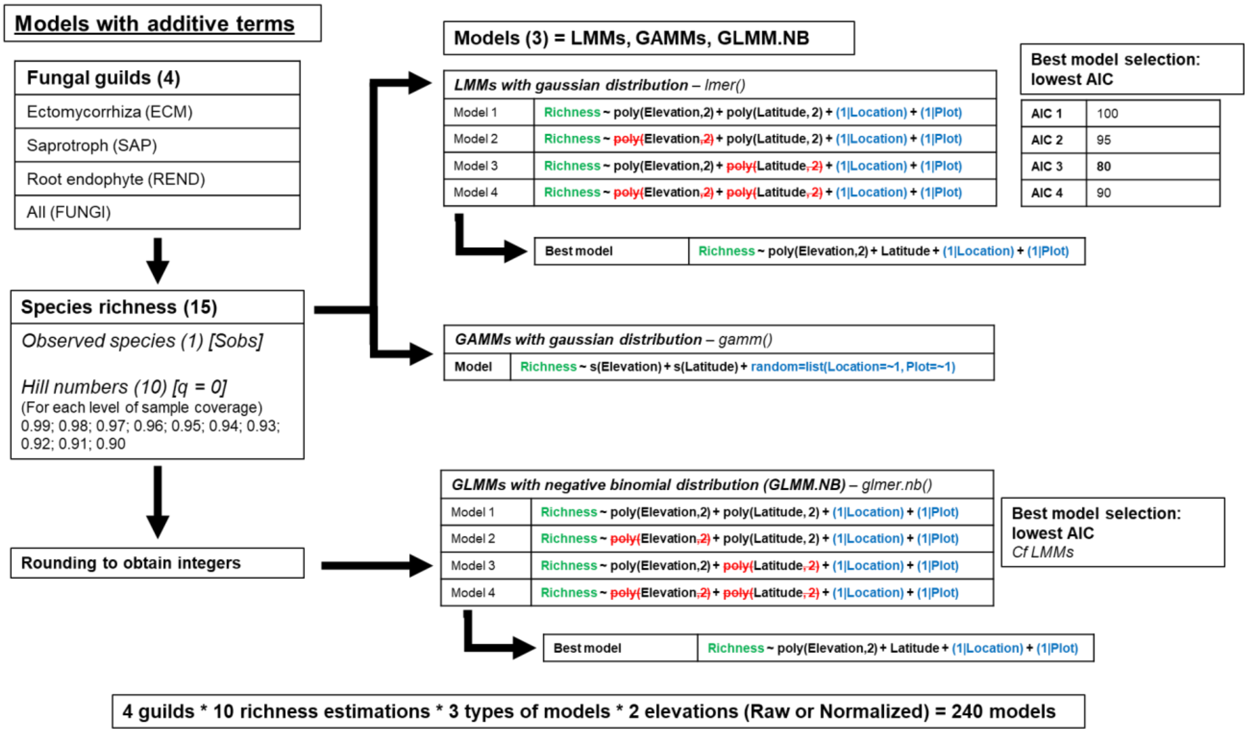

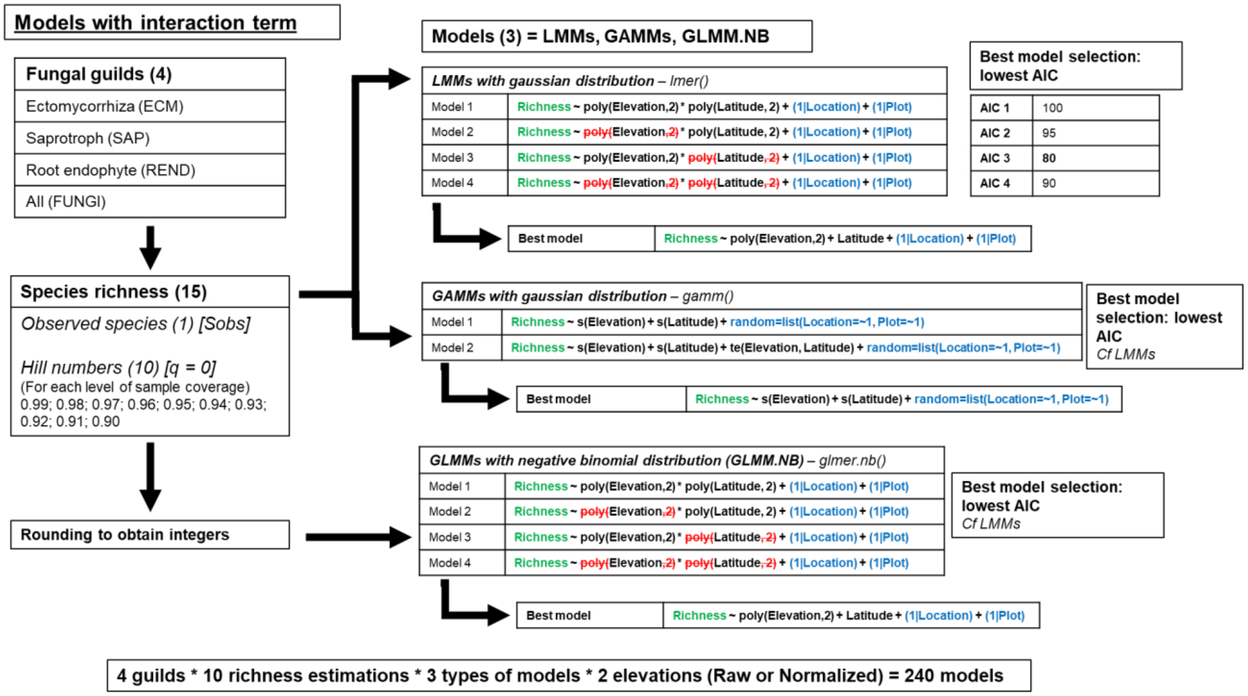

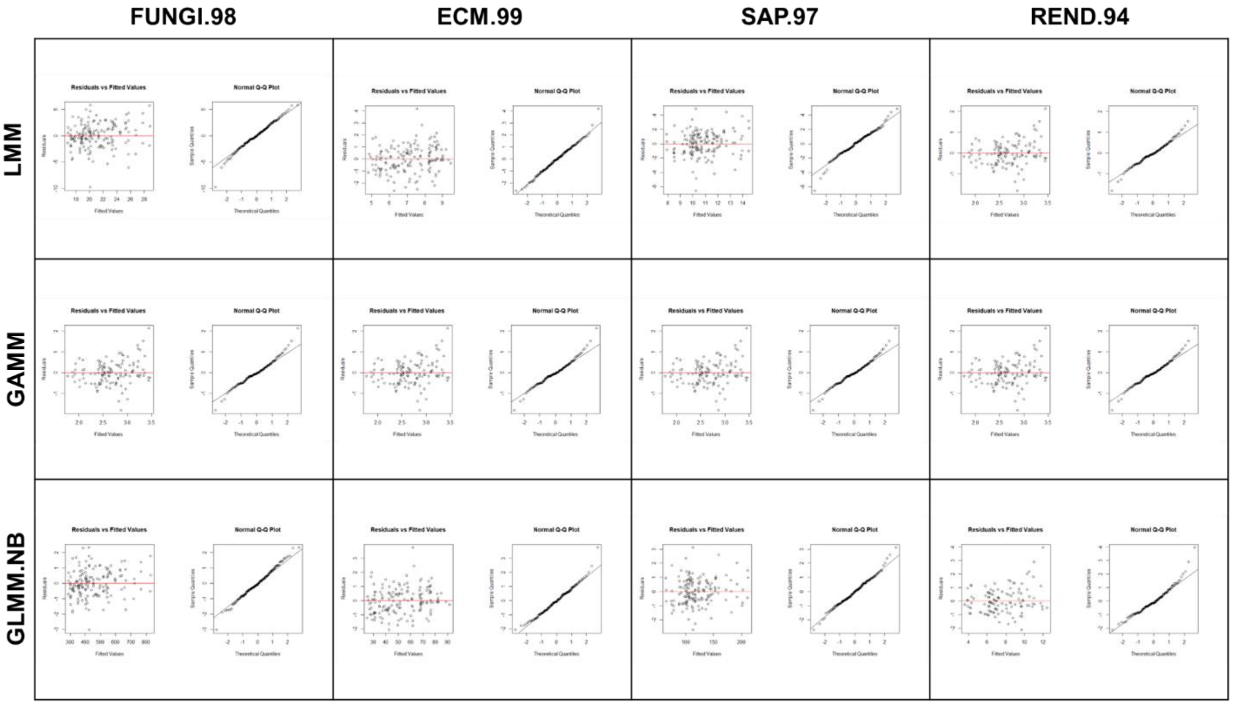
FUNGI.98
ECM.99
SAP.97
REND.94
LMM
GAMM
GLMM.NB
Residuals vs Fitted Values
Normal Q-Q Plot
